# Mendelian randomisation analysis to discover plasma metabolites mediating the effect of obesity on cancer risk

**DOI:** 10.1038/s41416-025-03170-7

**Published:** 2025-09-02

**Authors:** Thomas Yates, Molly Went, Charlie Mills, Philip Law, Ines Gockel, Carlo Maj, Johannes Schumacher, Claire Palles, Richard Houlston

**Affiliations:** 1 Division of Genetics and Epidemiology, The Institute of Cancer Research, Sutton, Surrey UK; 2 Department of Visceral, Transplant, Thoracic and Vascular Surgery, University Hospital of Leipzig, Leipzig, Germany; 3 Center for Human Genetics, University Hospital of Marburg, Marburg, Germany; 4 Institute of Cancer and Genomic Sciences, University of Birmingham, Birmingham, UK

**Keywords:** Cancer genomics, Cancer genomics

## Abstract

**Background:**

Obesity is a risk factor for several cancers, but the mechanistic basis is poorly understood. We sought to identify circulating metabolites mediating the effect of obesity on the risk of eight common cancers.

**Methods:**

Using European ancestry data, we applied two-sample Mendelian randomisation (2S-MR) to screen 856 plasma metabolites for associations with body mass index (BMI) and waist-hip ratio (WHR). Metabolite GWAS data were sourced from INTERVAL, and obesity traits from the GIANT consortium and UK Biobank. We assessed the impact of obesity-associated metabolites on cancer risk (384,738 cases across eight cancer types and 799,908 controls) and conducted mediation analyses to identify potential mediators of obesity-driven cancer risk.

**Results:**

MR analysis yielded 107 BMI-driven metabolites and 126 WHR-driven metabolites. The strongest relationships with cancer risk were between levels of obesity-driven 1-linoleoyl-GPC, 2-linoleoyl-GPC, 1,2-dilinoleoyl-GPC, 1-arachidonoyl-GPA, and 1-pentadecanoyl-2-linoleoyl-GPC and colorectal cancer (CRC). Additional associations were found between obesity-driven metabolites and breast cancer risk. Mediation analysis implicated multiple metabolites as potential mediators of obesity-driven CRC and breast cancer risk.

**Conclusions:**

As well as these findings highlighting how obesity-related metabolic changes influence cancer risk, our observations suggest potential interventional targets.

## Background

Obesity is a growing global health challenge, contributing not only to several major chronic conditions such as diabetes mellitus and cardiovascular disease, but increasingly is recognised as a risk factor for cancer [1]. The relationship between obesity and cancer is, however, complex and multiple pathways have been proposed as an underlying basis, including systemic inflammation and alterations in gut microbiota [2–4]. For example, in colorectal cancer (CRC), inflammation-related metabolic pathways have been implicated in mediating risk, such as the conversion of linoleate-containing phosphatidylcholines into arachidonate – a precursor of pro-inflammatory eicosanoids – a process regulated by the *FADS* gene cluster. In colorectal cancer (CRC), for instance, the *FADS* gene cluster has shown evidence of mediating cancer risk by metabolising linoleate-containing phosphatidylcholines into arachidonate [5–7].

A central challenge in studying obesity-related cancer risk lies in the accurate characterisation of obesity itself. Body mass index (BMI), a commonly used measure based on height and weight [8], does not differentiate between fat and lean mass. In contrast, waist-hip ratio (WHR) serves as a marker of central (abdominal) adiposity, capturing a distinct aspect of obesity associated with visceral fat accumulation [9]. Interpreting the causal role of obesity in cancer is further complicated by confounding lifestyle factors, such as alcohol consumption and smoking, which may co-occur with obesity and independently affect cancer risk.

One approach to gain insight into the mechanistic basis of obesity-related cancer risk is to identify circulating metabolites mediating the effects of obesity. The identification of mediator metabolites has the potential to provide insights into causal pathways and potentially provide targets for therapeutic intervention. While studies have supported the influence of obesity on levels of several plasma metabolites [10], since levels reflect complex biological processes, observational studies can be biassed by confounding factors and reverse causation. While the risk of several cancers have also been associated with levels of circulating metabolites [11], these associations are also subject to similar biases.

Mendelian randomization (MR) is an analytical approach which seeks to address these biases [12]. MR uses genetic variants as instrumental variables to evaluate the causal effects of exposures (risk factors) on outcomes. Since genetic variants are randomly allocated at conception and hence precede onset of disease, they are not influenced by reverse causation, and in the absence of pleiotropy (i.e. associations between genetic variants and disease through alternative pathways), they are largely independent of confounders.

Here, we have used MR (adhering to STROBE-MR best practices [13]) in conjunction with mediation analysis to identify plasma metabolites mediating the effect of obesity on the risk of eight common cancers (including 23 subtypes) – breast, prostate, CRC, lung, endometrial, oesophageal, renal cell carcinoma (RCC), ovarian – using data on 384,738 cases and 799,908 controls. By analysing both BMI and WHR, we have aimed to explore whether overall and central obesity exert differential effects on cancer risk through distinct metabolic pathways.

## Methods

**Fig. 1:**
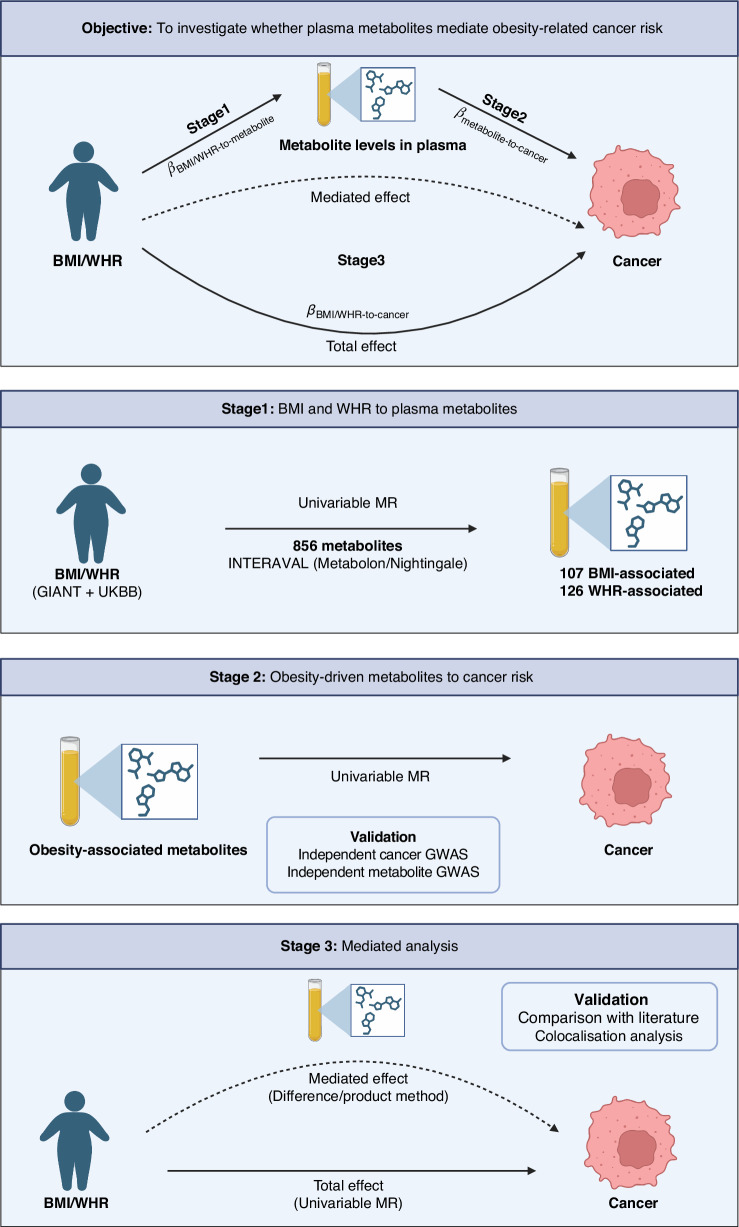
Study design. Stage 1: Metabolites associated with BMI/WHR are identified using univariable MR; Stage 2: Obesity-driven metabolites associated with cancer risk are identified using univariable MR; Stage 3: The effect mediated through metabolites is estimated using multivariable MR. Created with BioRender.com.

Figure 1 shows the study design [14]. Firstly, we estimated the effect of BMI and WHR on 856 plasma metabolites using MR. Secondly, we estimated the effect of BMI/WHR-driven metabolites on cancer risk, again using MR. Thirdly, statistically significant associations between metabolites and cancer risk were prioritised by Bayesian colocalisation and we performed mediation analyses to elucidate the metabolic mediators of the relationship between obesity with cancer risk.

### Stage 1: BMI and WHR to plasma metabolites

We estimated the effect of BMI and WHR on circulating plasma metabolite levels (*β*_BMI/WHR-to-metabolite_ in Fig. 1) using two-sample MR (2S-MR) [15]. Two-sample MR can estimate the causal effect of the exposure on the outcome using summary statistics from genome-wide association studies (GWAS) when three core assumptions are made about the instrumental variables (IVs) – the IVs are associated with the exposure, there are no confounders of the IV-outcome associations, and the IVs only influence the outcome through the exposure.

GWAS were selected to maximise sample sizes whilst avoiding sample overlap. For BMI and WHR, we used GWAS meta-analysis data on individuals of European ancestry from the GIANT consortium and the UK Biobank (BMI, 681,275 samples; WHR, 697,734 samples) [16, 17]. Consortium details are provided in Supplementary Table 1 and post-hoc power calculations [18] provided in Supplementary Tables 2 and 3. For the GWAS of plasma metabolite levels, we used data from the INTERVAL study [19], which quantified 867 plasma metabolites in individuals of European ancestry using Metabolon (726 metabolites) and Nightingale (141 metabolites) assays that measured plasma abundances in 8153 and 37,359 individuals, respectively [20].

The effect of BMI and WHR on plasma metabolite levels was estimated using the inverse variance weighted random-effects (IVW-RE) model [21]. The IVs for BMI/WHR were found using PLINK v.1.9 [22, 23] (*P*  <  5 × 10^−8^; r^2^  <  0.01, within a 0.5 Mb region) to clump genome-wide significant single nucleotide polymorphisms (SNPs) with a minor allele frequency (MAF) > 0.01 referenced to the 1000 Genomes Project European panel (phase 1 integrated release 3 March 2012) [24] (Supplementary Tables 4 and 5). For each linkage disequilibrium (LD) block, the IV was chosen as the SNP with the smallest *P*-value. SNPs in the human major histocompatibility complex region (chr6:28,477,897–33,448,354; GRCh37) were removed from the datasets before clumping, due to the likelihood of horizontal pleiotropy. Data harmonisation and MR analyses were conducted using TwoSampleMR v.0.5.9 [25, 26], with SNPs not present in both the exposure and outcome GWAS, with ambiguous alleles, or palindromic with a MAF > 0.42 removed. For metabolites present in both Metabolon and Nightingale assays, data from the Nightingale GWAS was used due to its larger sample size. A Bonferroni-corrected *P*-value threshold was used to define statistical significance, adjusting for the number of harmonised metabolites. Many of the metabolites within the datasets were non-independent, making this correction excessively strict; however, we sought to minimise false positives. We used *F*-statistics [27], which is a measure of the strength of the association of IVs to test for weak instrument bias. In accordance with previously published work [28] we considered a *F-*statistic <10 as being indicative of weak instrument bias. Using Cochran’s *Q* statistic, we considered a *P*-value  <  0.05 as reflecting significant heterogeneity. To ensure robustness of any associations, MR analyses using the inverse variance weighted fixed-effects, maximum likelihood, simple median, weighted median, simple mode, and weighted mode were also performed [29, 30]. Leave-one-out analysis was also performed to detect outlying and pleiotropic SNPs [31]. By removing each SNP from the MR analysis sequentially, if the association is no longer nominally significant (*P* > 0.05) with the SNP removed, this indicates that a particular SNP is driving the association. The MR-Egger intercept test [32] was used to evaluate directional pleiotropy when three or more IVs were available. We considered non-negligible directional pleiotropy to be present when the MR-Egger intercept was not null (i.e. *P*_Egger-intercept_  <  0.05). We also assessed reverse causation, wherein the effect of cancer may influence plasma metabolite levels, using bidirectional MR. Bidirectional MR was undertaken following the same steps as above, with the exposure and outcome switched. If significant associations were detected by both forward and reverse MR analysis of an exposure-outcome pair, reverse causation could not be discounted. Exposure-outcome pairs that failed any of the sensitivity tests were excluded from further analysis.

### Stage 2: Obesity-driven metabolites to cancer risk

Next, we performed 2S-MR to estimate the causal effects of the obesity-driven metabolites on the risk of each of the eight cancers (*β*_metabolite-to-cancer_ in Fig. 1). For this analysis, we used summary cancer GWAS effect estimates from: (1) Online consortia resources, for breast (BCAC; https://bcac.ccge.medschl.cam.ac.uk/, accessed July 2022) and prostate cancer (PRACTICAL; http://practical.icr.ac.uk/; accessed July 2022); (2) GWAS Catalog (https://www.ebi.ac.uk/gwas/), for ovarian, CRC, endometrial, and lung cancers (accessed September 2022); (3) Investigators of published work, for RCC and oesophageal cancer (Supplementary Table 6). Since the UK Biobank was used to obtain genetic instruments for obesity traits, the CRC and oesophageal GWAS association statistics were recalculated from primary data excluding UK Biobank samples to avoid sample overlap bias. IVs of the metabolite levels were defined as in Stage 1 (Supplementary Table 7). SNPs were harmonised and proxy SNPs (r^2^ > 0.8, within a 0.5 Mb region) were used for obesity-driven metabolites that had no suitable IVs after harmonisation. Proxy SNPs were identified using the 1000 Genomes Project European panel (phase 1 integrated release 3 March 2012) [24]. Cancer subtype summary statistics were available for lung, breast, and ovarian cancers. FinnGen GWAS cohort data (https://www.finngen.fi/en; release R10) were used for the discovery phase of CRC subtypes and validation of CRC, breast, and prostate cancer associations. Where available, metabolite GWAS data from the Canadian Longitudinal Study of Aging (CLSA) [33] were used for validation of metabolite and cancer risk associations, as per Stage 1. Associations between metabolites and cancer risk that failed validation were not taken forward.

Using these data, we carried out 2S-MR to investigate the effects of obesity-driven plasma metabolite levels on cancer risk. The effects were estimated as odds ratios (OR) per standard deviation (S.D.) increase in a metabolite level (OR_SD_), with a 95% confidence interval (CI). We used the IVW-RE method for metabolites with two or more instrumental variables and the Wald ratio method [34] for those with a single IV. The same sensitivity analyses as per Stage 1 were used to detect directional pleiotropy and heterogeneity, with metabolite-cancer pairs failing any of the sensitivity analyses excluded from further analysis.

We performed colocalisation analysis using the coloc R package v.5.2.3 [35] to examine whether the genetically predicted metabolite level and cancer risk shared the same causal variant. In particular, we performed enumeration colocalisation analysis using approximate Bayes factors assuming there was, at most, one causal variant per trait. This method calculates the posterior probability of: H_0_, neither trait has a genetic association in the region; H_1_, only the exposure has a genetic association in the region; H_2_, only the outcome has a genetic association in the region; H_3_, both traits are associated, but with different causal variants; H_4_, the exposure and outcome are associated and share a single causal variant. In this analysis, all SNPs within a +/−0.5 Mb region around the SNP acting as an IV for the metabolite were included. To adjust for the number of SNPs within each locus [36], the prior probabilities were chosen as p_1_ = p_2_ = 1/(10× number of SNPs) and p_12_ = p_1_/10, where p_1_, p_2_, and p_12_ are the prior probabilities that a particular SNP within the locus is only associated with trait 1, trait 2, or both traits, respectively. The posterior probability of H_4_ or *PP*_shared_ (two traits sharing a single causal variant) > 0.8 was considered to provide evidence of colocalisation. Sensitivity analysis was performed by observing how *PP*_shared_ varies as the value of p_12_ is changed; however, no exposure-outcome pairs were removed from further analysis as a result of the sensitivity analysis.

### Stage 3: Mediation analysis

We undertook mediation analysis to calculate the proportion of the effect of each of the obesity traits on cancer risk, potentially mediated by the obesity-driven metabolites, using the difference method [37, 38]. This estimates the combined mediated effect of the metabolites on the obesity-driven cancer risk. The obesity and metabolite IVs must be reclumped together; however, if the sample sizes of the obesity and metabolite GWAS were significantly different, it is possible that only IVs from the GWAS with the larger sample size were retained, leading to weak instrument bias in the IVs of the other trait. Where weak instrument bias prevented estimation of the combined mediated effect, we used the product method [39], whereby the metabolite’s mediated effects were estimated individually, using the same IVs from Stages 1 and 2. However, this precluded estimation of the combined mediated effect for non-independent mediators. The same exposure and outcome GWAS from Stage 1 and Stage 2 were used for both univariable and multivariable MR analyses.

The World Cancer Research Fund and the American Institute for Cancer Research have concluded that there is strong evidence that alcohol intake, obesity, physical activity, and diet affect the risk of developing cancer [40]. Therefore, the IVs of the metabolite mediators were manually checked for these pleiotropic associations using data from the GWAS Catalog [41]. If a pleiotropic association was present, the IV was removed and the analysis repeated. Potential obesity-mediating metabolites that did not have the same direction of effect as the total effect of BMI/WHR on cancer risk (i.e. sgn(*β*_BMI/WHR-to-metabolite_ × *β*_metabolite-to-cancer_) ≠ sgn(*β*_BMI/WHR-to-cancer_)) were also excluded from mediation analysis.

For the difference method, we used univariable MR to estimate the total effect of BMI/WHR on cancer risk (*β*_BMI/WHR-to-cancer_ in Fig. 1) and multivariable MR to estimate the direct effect of BMI/WHR on cancer risk. IVs for the univariable MR analysis were selected as in Stage 1. IVs for the multivariable MR analysis were selected by reclumping the union of the obesity measure and metabolite from Stages 1 and 2. The reclumping used the same method and values as in Stage 1 but chose the IV as the SNP with the smallest *P*-value in any of the GWAS. The metabolite-mediated effect (i.e. the effect of BMI/WHR on cancer risk accounted for by the associated metabolites) was calculated as the difference between the total and direct effects. The proportion of the total effect of BMI/WHR on cancer risk mediated by the metabolite was estimated by dividing the metabolite-mediated effect by the total effect. Multivariable MR data harmonisation and clumping was performed using the mv_extract_exposures_local and mv_harmonise_data functions in TwoSampleMR v.0.6.2 using the 1000 Genomes Project European reference panel (phase 1 integrated release 3 March 2012) [24], whilst multivariable MR analyses were performed using the qhet_mvmr function in MVMR v.0.4 [42]. To estimate the 95% confidence interval for the multivariable MR analysis of BMI and breast cancer risk, 100 bootstrap iterations were used as this was the maximum number of iterations feasible with available computational resources. The confidence interval for BMI and CRC risk could not be calculated using qhet_mvmr due to computational constraints. Therefore, for BMI and CRC risk the direct effect estimate and confidence interval were also calculated using the ivw_mvmr function, which does not attempt to correct for weak instrument bias and pleiotropy. Conditional *F*-statistics and horizontal pleiotropy estimates were calculated using strength_mvmr and pleiotropy_mvmr, respectively, within MVMR v.0.4. The threshold for weak instrument bias was defined as conditional *F-*statistic < 10 [42], and we considered a *P*-value  <  0.05 from the modified form of Cochran’s Q statistic as being indicative of significant heterogeneity. If weak instrument bias or pleiotropy was detected, we instead used the product method. Phenotypic correlation matrices were calculated using the estimateSyy function in metaCCA v.1.13.2 [43], and genetic covariance matrices were calculated using the phenocov_mvmr function in MVMR v.0.4.

For the product method, the metabolite-mediated effect was instead calculated using the univariable MR results from Stage 2 as *β*_BMI/WHR-to-metabolite_ × *β*_metabolite-to-cancer_. The proportion mediated was estimated as in the difference method. An additional constraint of non-overlapping IVs between the exposure and mediator must be fulfilled when using univariable MR in mediation analysis. Any SNP that is used as an instrument for both the exposure and mediator will be pleiotropic when estimating *β*_BMI/WHR-to-metabolite_ [44]. Therefore, the IVs for the exposure and mediators were checked to ensure no overlapping IVs (defined as r^2^  <  0.01, within 0.5 Mb of each other) were present. Overlapping IVs were removed from the GWAS with the larger sample size to minimise the loss of power.

We contextualised metabolites into their biological pathways by referencing the Kyoto Encyclopedia of Genes and Genomes (KEGG) database [45]. To examine the relationship between IV genotype and gene expression we performed an expression quantitative trait loci analysis using the Genotype-Tissue Expression (GTEx) Portal [46, 47].

## Results

### Stage 1: BMI and WHR to plasma metabolites

**Fig. 2:**
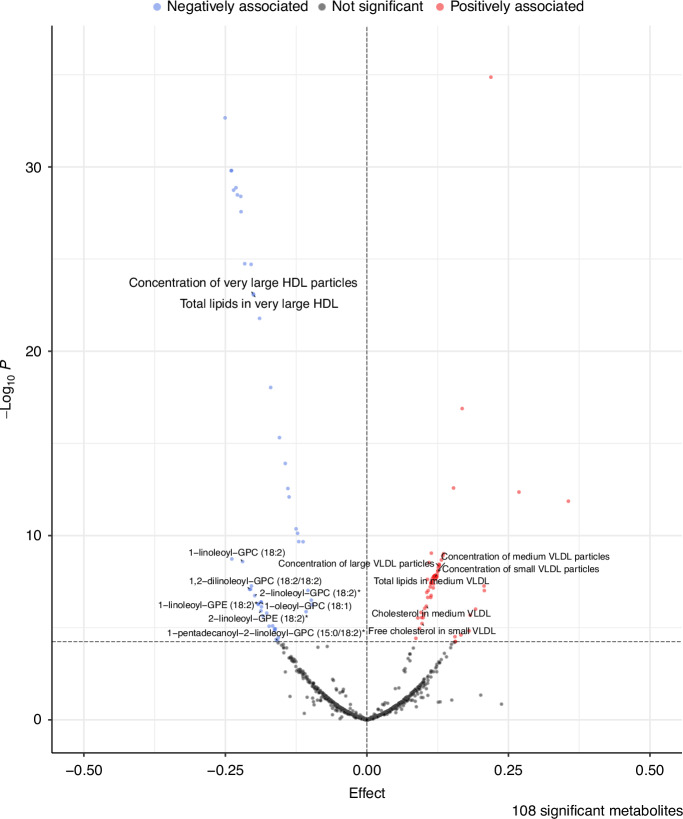
Volcano plot of potentially causal associations between BMI and plasma metabolites. The dashed horizontal line indicates the Bonferroni-corrected significance threshold (*P* = 5.84 × 10^−5^) and all metabolites significantly associated with BMI in the MR analysis are coloured. Metabolites that are negatively associated with BMI are coloured blue, whilst those positively associated with BMI are coloured red. The dashed vertical line indicates a null effect. Solid lines are used to connect labels to data points. The total number of significant metabolites is shown in the bottom-right corner. Created using the EnhancedVolcano (v1.20.0) R package [59]. HDL high-density lipoprotein, VLDL very-low-density lipoprotein, GPC glycero-phosphatidylcholine, GPE glycero-phosphatidylethanolamine.

After harmonisation, 856 plasma metabolite levels were used as outcomes. The *F*-statistics of the obesity measures were greater than 59.8 for BMI and 45.8 for WHR; hence there was no evidence of weak instrument bias [28] (Supplementary Tables 2 and 3). Furthermore, we had >80% power to detect a relationship for all the BMI/WHR-metabolite pairs, provided OR_SD_ was at least 1.05. Of the metabolites screened, 108 were estimated to be influenced by BMI and 128 by WHR, using a Bonferroni-adjusted threshold of *P* < 5.84 × 10^−5^ (0.05/856), highlighting the For influence of BMI and WHR on levels of plasma metabolites (Fig. 2 and Supplementary Fig. 1 and Supplementary Tables 8 and 9). We did not find significant heterogeneity for Bonferroni-significant metabolites (*P* < 0.05), apparent directional horizontal pleiotropy using the MR-Egger test (*P*_Egger-intercept_ <  0.05), or a single SNP driving any of the associations from the leave-one-out analysis. One BMI-associated metabolite and two WHR-associated metabolites showed significant bidirectional effects and were removed from further investigation (Supplementary Tables 10 and 11). After performing these analyses, 107 BMI-driven metabolites (28 unique to BMI) and 126 WHR-driven metabolites (48 unique to WHR), for a total of 154 obesity-driven metabolites, were identified with no apparent heterogeneity, directional pleiotropy or reverse causation.

### Stage 2: Obesity-driven metabolites to cancer risk

**Fig. 3:**
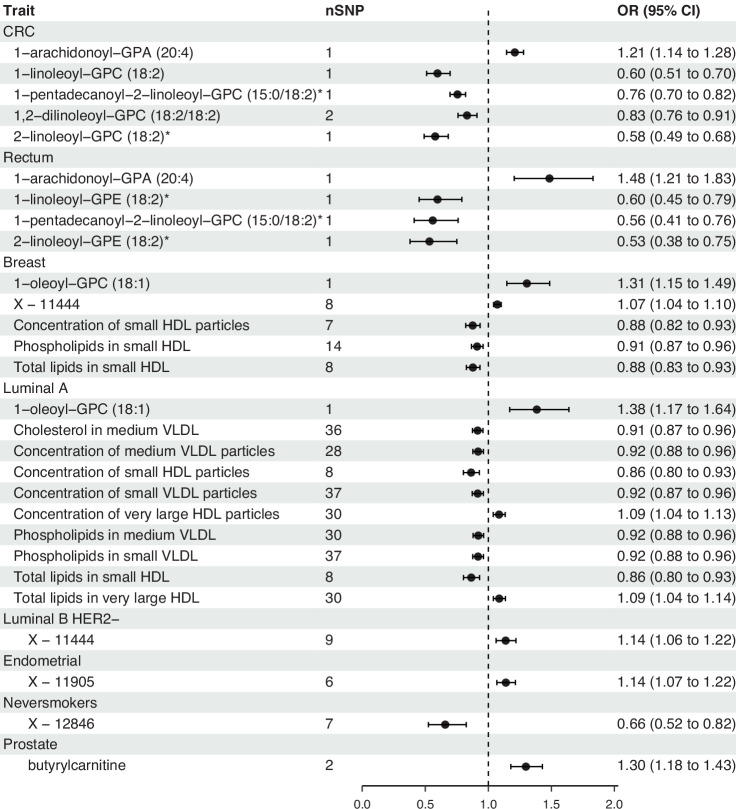
Forest plot of obesity-driven metabolites associated with cancer risk. Odds ratios and 95% confidence intervals were estimated using MR utilising either the IVW-RE method or Wald ratio (depending on the number of IVs). The names of the metabolites are given in the left column, stratified by the associated cancer risk. The vertical dashed line indicates a null effect. The error bars show 95% confidence intervals around the estimated odds ratio. nSNP number of SNPs. Created using the forester (v0.3.0) R package [60]. HDL high-density lipoprotein, VLDL very-low-density lipoprotein, GPC glycero-phosphatidylcholine, GPE glycero-phosphatidylethanolamine, GPA glycero-phosphate.

For 81% of the metabolites carried forward from Stage 1 we had >80% power to detect a relationship, provided OR_SD_ was >1.25. Based on a Bonferroni-adjusted threshold, MR revealed that a per S.D. reduction in genetically predicted levels of three BMI-associated metabolites: 2-linoleoyl-GPC, 1,2-dilinoleoyl-GPC, 1-pentadecanoyl-2-linoleoyl-GPC, and BMI- and WHR-associated 1-linoleoyl-GPC were associated with increased risk of CRC (Fig. 3 and Supplementary Tables 12 and 13). One S.D increase in BMI-associated 1-arachidonoyl-GPA was also associated with increased risk of CRC. Three BMI-associated metabolites were identified as significantly associated with reduced risk of rectal cancer: 1-linoleoyl-GPE, 2-linoleoyl-GPE, and 1-pentadecanoyl-2-linoleoyl-GPC. One S.D increase in BMI-associated 1-arachidonoyl-GPA was also associated with increased risk of rectal cancer. One S.D. increase in genetically predicted levels of BMI-associated 1-oleoyl-GPC and WHR-associated X-11444 were associated with increased breast cancer risk. One S.D. reduction in WHR-associated concentration of small HDL particles, total lipids in small HDL, and phospholipids in small HDL were also associated with breast cancer risk. All the BMI/WHR-driven metabolites associated with breast cancer (excluding phospholipids in small HDL) were also found to be significantly associated with, and have the same direction of effect in, at least one subtype of breast cancer. Furthermore, a per S.D. reduction in multiple genetically predicted measures of very-low-density lipoprotein levels were associated with increased luminal-A breast cancer risk. Finally, one S.D. decrease in levels of WHR-associated X-12846 was associated with increased lung cancer risk in never-smokers and one S.D. increase in levels of BMI-associated butyrylcarnitine was associated with increased prostate cancer risk. WHR-associated X-11905 was nominally associated with increased endometrial cancer risk; however, a single outlying SNP driving the association was identified during the leave-one-out analysis and this metabolite-cancer pair was not considered for further analysis. None of the obesity-driven metabolites were found to be significantly associated with RCC, oesophageal, or ovarian cancer.

**Fig. 4:**
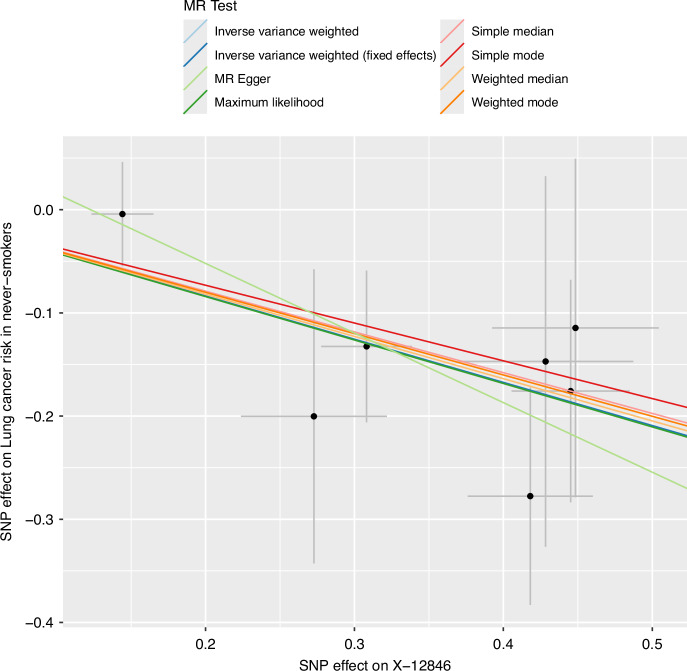
Example scatter plot for a metabolite-cancer association showing consistent direction of effect for all MR methodologies. Each line represents a different MR methodology with the slope of the line indicating the estimated effect size. Error bars indicate the standard error of the association between the SNP and cancer risk (vertical) or the SNP and levels of the metabolite (horizontal). Created using the TwoSampleMR (v0.5.9) R package [25, 26].

All significant associations had consistent direction of effect under the different MR methodologies (Fig. 4 and Supplementary Table 12). To further test whether plasma levels of the obesity-driven metabolites were causal for CRC, breast cancer, and prostate cancer risk, we performed validation MR analyses using cancer GWAS data from the FinnGen cohort. Estimates were consistent with a causal effect of each of the five obesity-driven metabolites on CRC risk (Bonferroni-adjusted threshold *P* < 1.00 × 10^−2^) (Supplementary Fig. 2 and Supplementary Table 14); however, using the breast cancer GWAS from the FinnGen cohort, four of the obesity-driven metabolites were not validated and the final metabolite had no instruments post harmonisation of data. The association between butyrylcarnitine and prostate cancer was not validated in the FinnGen cohort. Although there was potential sample overlap between the independent cancer GWAS and the GWAS of the FinnGen cohort for breast and prostate cancers, the metabolites were sufficiently powered (all *F*-statistics >100; Supplementary Table 13) after harmonisation with the FinnGen cancer GWAS to avoid inflated weak instrument bias [48]. Validation MR analyses were not performed for the other metabolite-cancer associations due to lack of appropriate endpoints in the FinnGen cohort. Validation MR analyses were also performed for metabolites with GWAS data available from the CLSA cohort using both the independent and FinnGen cancer GWAS (Supplementary Table 15). The association between 1-oleoyl-GPC and breast cancer reached significance in the independent cancer GWAS (BCAC), but not in the FinnGen cancer GWAS. The lack of a significant association in the FinnGen cancer GWAS may be explained by the limited power to detect an association as compared with analysis based on the CLSA and BCAC GWAS. Hence, we did not preclude this metabolite-cancer pair from further analysis. None of the associations showed significant bidirectional effects (Supplementary Table 16). We also performed colocalisation analysis on the 27 metabolite-cancer pairs, finding 14 pairs with at least one IV that met the H_4_ threshold (*PP*_shared_ > 0.8; Supplementary Figs. 4–17). A total of 12 metabolite-cancer associations passed all sensitivity analyses and were taken forward to Stage 3 (Supplementary Table 17).

### Stage 3: Mediation analysis

The associations between BMI/WHR and luminal-A breast cancer and BMI and rectal cancer did not meet the Bonferroni threshold (*P* < 8.33 × 10^−3^) and were not taken forward in the mediation analysis (Supplementary Table 18).

We used both mediation methods (see Methods) to estimate the metabolite-mediated effect. All mediation analyses using the difference method suffered from weak instrument bias in the multivariable MR analysis (Supplementary Table 19). This was due to all but one of the metabolites being reliant on a single IV during initial clumping, compared to the hundreds of IVs for BMI/WHR. During reclumping of the combined BMI/WHR and metabolite IVs, all but one of these single metabolite IVs were removed, with most remaining IVs only weakly associated with the metabolites. Horizontal pleiotropy was also detected for all multivariable MR analyses. Using the qhet_mvmr function within the MVMR R package, direct effect estimates can be calculated, adjusted for the violated MR assumptions; however, this analysis provided no evidence of any significant metabolite-mediated effect (Supplementary Table 20).

**Fig. 5:**
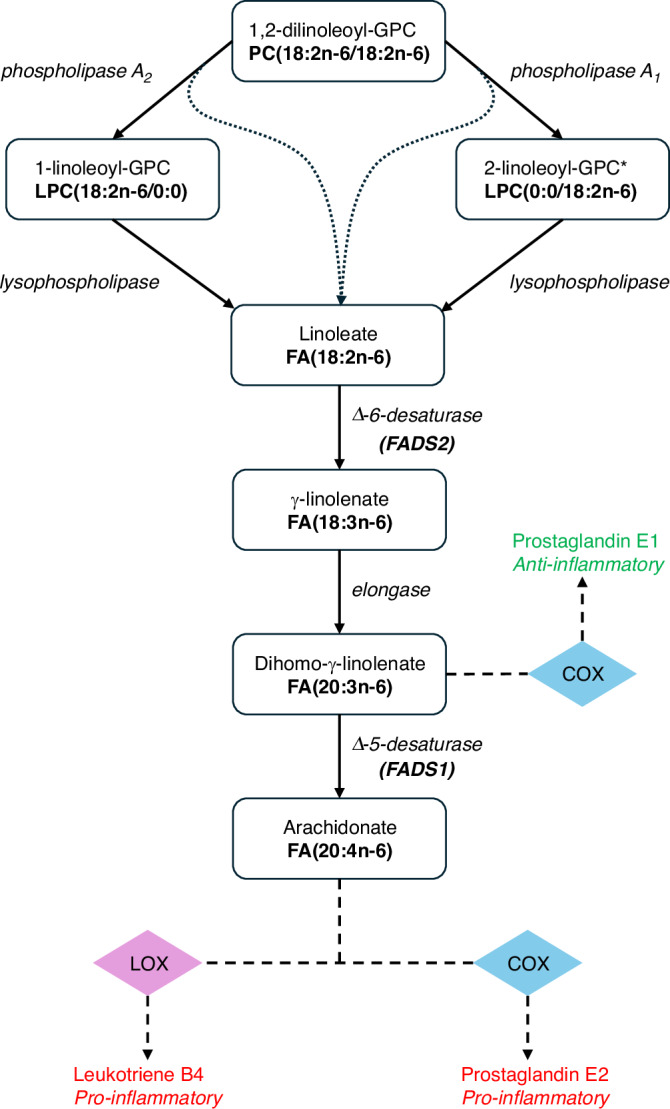
Omega-6 polyunsaturated fatty acid metabolic pathway. An illustration of the omega-6 polyunsaturated fatty acid metabolic pathway with information taken from the KEGG database [45]. The arrows show individual metabolic reactions. The enzymes for each reaction are shown in italics. The dotted lines show reactions that produce linoleate as a by-product. The dashed lines show the processes by which inflammatory mediators are produced. The metabolite abbreviations are shown in bold. The enzymatic process catalysed by Δ-5-desaturase (*FADS1*) mediates the ratio of pro- to anti-inflammatory lipids present. PC phosphatidylcholine, LPC lysophosphatidylcholine, GPC glycero-phosphatidylcholine, FA fatty acid, COX cyclooxygenase, LOX lipoxygenase.

For the product method, estimates for the mediated effect of each individual metabolite were calculated; however, a combined effect could not be calculated as the metabolites are all localised in the omega-6 polyunsaturated fatty acid (PUFA) metabolic pathway (Fig. 5) and are therefore not independent. Each metabolite’s mediated effect will therefore also contain contributions from associated metabolites and summing all metabolites’ mediated effects will lead to counting contributions more than once [44]. All mediators had a significant mediated effect after Bonferroni correction (*P* < 1.25 × 10^−2^ (0.05/4)) (Supplementary Table 21). For CRC risk, three BMI-associated metabolites: 2-linoleoyl-GPC, 1,2-dilinoleoyl-GPC, and 1-pentadecanoyl-2-linoleoyl-GPC, showed a mediated effect of at least 13%. Similarly, for breast cancer, BMI-associated 1-oleoyl-GPC showed a mediating effect in excess of 29%. No IVs for metabolites mediating either CRC risk or breast cancer risk showed pleiotropic associations in the GWAS Catalog [41] (Supplementary Table 22).

### Role of fatty acid desaturase genes in obesity-driven CRC risk

Accepting the limitations of the mediation analysis and although speculative, the omega-6 polyunsaturated fatty acid metabolic pathway may explain the CRC risk associations found by the MR analyses. We found that genetically predicted lower levels of 2-linoleoyl-GPC, 1,2-dilinoleoyl-GPC, and 1-pentadecanoyl-2-linoleoyl-GPC were associated with an increased risk of CRC, which expanded the causal evidence of linoleate-containing phosphatidylcholine (PC) metabolites with decreased CRC risk [5, 6]. Furthermore, all nominally significant linoleate-containing PC metabolites were negatively associated with CRC risk and all nominally significant arachidonate-containing PC metabolites, as well as arachidonate itself, were positively associated with CRC risk (Supplementary Fig. 3). These two omega-6 PUFAs are found on opposite sides of the *FADS1* Δ-5-desaturase enzymatic step within the omega-6 PUFA pathway (Fig. 5), implicating *FADS1* expression as the underlying factor determining CRC risk.

The single IVs for 2-linoleoyl-GPC and 1-pentadecanoyl-2-linoleoyl-GPC are all located in, or near to, the *FADS* gene cluster, whilst one of the two IVs for 1,2-dilinoleoyl-GPC is located in *FADS1*. Furthermore, these IVs are significant *FADS1/2* cis-expression quantitative trait loci (eQTLs) in colon tissue [46], with opposite direction of effect for each gene. Where gene expression data for a particular IV was unavailable, a SNP in strong LD (r^2^ > 0.9) was used as a proxy. Colocalisation analysis found that all three metabolites showed evidence of a shared causal variant (*PP*_shared_ > 0.8) with *FADS1* in sigmoid colonic tissue, but not transverse colonic tissue (Supplementary Table 23 and Figs. 18–20). The reported most likely causal variant for all but one metabolite was rs1535, which was also significantly associated with CRC risk in the cancer GWAS. However, none of the GWAS significant SNPs for BMI/WHR showed evidence of a shared causal variant (*PP*_shared_ > 0.8) with any *FADS* gene in colon tissue (Supplementary Tables 24 and 25).

## Discussion

To advance our understanding of obesity-mediated cancer risk, we performed a discovery metabolome-wide MR analysis for eight common cancers. To our knowledge, our study is the first to comprehensively evaluate potential causal associations from plasma metabolites for multiple cancers. A major strength of our study has been the adherence to the three core assumptions of MR. Assumption 1 (relevance) was addressed by including only exposures with *F*-statistics >10 to minimise weak instrument bias. While this is conservative approach and reduces false positives it may have excluded biologically relevant metabolites. Assumption 2 (independence) was addressed by restricting analyses to individuals of European ancestry to reduce population stratification. Hence, future studies are required to establish whether the same metabolites mediate the effect of obesity in populations of non-European ancestries. Furthermore, we were unable to explore sex-specific effects due to the lack of sex-stratified data. Assumption 3 (exclusion restriction) was satisfied by excluding exposure–outcome pairs failing sensitivity analyses and removing IVs with known pleiotropic associations from the GWAS Catalog.

Accepting these caveats, we identify potential mediators of obesity-driven cancer risk, with three plasma metabolites from univariable MR that were associated with both BMI and CRC risk and one metabolite associated with BMI and breast cancer. While mediation analysis was consistent with the effect of obesity on CRC and breast cancer being potentially mediated by these metabolites we highlight, inevitably, the wide confidence intervals reflect the limited predictive power of the metabolite IVs. Furthermore, mediated effect estimates for a single metabolite may be overinflated because of indirect contribution from other associated factors [44].

A contemporaneous study investigating the causal effect of plasma metabolites on CRC risk found evidence to support a relationship between linoleate- and arachidonate-containing PC metabolites and CRC risk [5]. Furthermore, 1,2-dilinoleoyl-GPC and 1-linoleoyl-2-linoleoyl-GPC, another precursor of 2-linoleoyl-GPC, were found to be mediators of obesity-driven CRC risk. Although using multivariable MR for the mediation analysis, no conditional *F*-statistics or pleiotropy estimates were reported. Hence, our observations are in broad agreement with this study’s findings, which reported a metabolite-mediating effect of 61% for BMI-driven CRC risk.

Increased levels of oleate, a downstream product of 1-oleoyl-GPC, have shown a conflicting effect on breast cancer risk and evolution, depending on subtype and menopausal status [49, 50]. Furthermore, obesity is known to be protective for premenopausal women, but a risk factor for postmenopausal women [4, 51]. This makes it difficult to speculate on potential pathways by which obesity-driven breast cancer risk is mediated by the metabolome, due to the lack of menopausal-stratified breast cancer GWAS available for this study. A study using a menopausal-stratified breast cancer GWAS may therefore detect other mediators, which we unfortunately were not empowered to identify.

Although further research is needed to verify our findings and elucidate the underlying biological mechanism, our results are consistent with a model by which obesity mediates CRC risk by generating a proinflammatory state [2, 52]. Specifically, arachidonate is metabolised by COX-2 to produce inflammatory mediators including prostaglandin E2, which affects CRC carcinogenesis [53]. Moreover, this accords with *FADS2* being a risk locus for CRC [54] and the ability of aspirin to irreversibly inhibit COX-1 and COX-2 and lower proinflammatory signals for CRC chemoprevention [55]. The *FADS1/2* genes are also overexpressed in colon adenocarcinomas [56], further emphasising the role of the *FADS* genes in CRC risk. While plasma levels of arachidonate have been reported to be associated with an increased risk of CRC [6, 7], the *FADS* gene cluster is known to be a region of high LD [57], hence the pleiotropy makes it difficult to definitively resolve which PUFA metabolites are driving the genetically predicted association with CRC risk.

Using both BMI and WHR to assess obesity, we compared their effects on the plasma metabolome. WHR was associated with a greater number of, and more unique, metabolite associations than BMI, despite fewer IVs and similar sample sizes. Since both metrics showed comparable proportions of Metabolon-assay associations this excludes assay heterogeneity and suggests WHR may better capture obesity-related metabolic changes and disease risk, in keeping with previous findings [9].

Herein we have been able to provide evidence for potential mediation of obesity-driven CRC and breast cancer risk by performing a metabolome-wide MR analysis. Considering that obesity is a highly polygenic trait with more than 500 associated loci influencing over 150 metabolites, it is unlikely that any single metabolite will explain a high proportion of the effect of obesity-related traits on risk of any specific cancer. Moreover, while the risk of several cancers have been documented to be strongly influenced by obesity, notably CRC [58] and breast cancer [51], it is not necessarily the case that mediators of obesity-related cancer risk will be consistent across cell lineages. Furthermore, in the case of breast cancer, obesity has been documented to be both protective or a risk factor depending upon menopausal status [4]. Hence, further MR-based analyses using additional omics data in conjunction with larger cancer GWAS datasets have the potential to definitively ascertain which metabolites mediate obesity-driven cancer risk.

## Supplementary information

Supplementary Figures

Supplementary Tables

STROBE-MR Checklist

## Data Availability

Instrumental variables are given in Supplementary Tables 4, 5, and 7. Summary GWAS BMI/WHR data are available from https://portals.broadinstitute.org/collaboration/giant/index.php/GIANT_consortium_data_files. Summary GWAS metabolite data are available from https://www.omicspred.org/. Summary GWAS cancer data are available from: https://bcac.ccge.medschl.cam.ac.uk/bcacdata/ (breast cancer); http://practical.icr.ac.uk/blog/?page_id=8088 (prostate cancer); GWAS Catalog ID: GCST004481 (ovarian cancer); GWAS Catalog ID: GCST006465 (endometrial cancer); GWAS Catalog ID: GCST004748 (lung cancer); direct communication with consortia (renal and esophageal cancers); - phs001415.v1.p1, phs001315.v1.p1, phs001078.v1.p1, phs001903.v1.p1, phs001856.v1.p1 and phs001045.v1.p1 (US based studies) and GWAS Catalog ID: GCST90129505 (European based studies) colorectal cancer. FinnGen data can be accessed by following the instructions at https://www.finngen.fi/en/access_results. Source data are provided within the supplementary data of this paper. Code used to generate the results presented in this paper is available online at 10.5281/zenodo.15836592 and https://github.com/houlstonlab/obesity-metabolite-MR.
