# Supplementary material for: Mendelian randomisation analysis to discover plasma metabolites mediating the effect of obesity on cancer risk
Source: Br J Cancer. 2025 Sep 2;133(9):1344–53. doi: 10.1038/s41416-025-03170-7 (PMC12572173; doi:10.1038/s41416-025-03170-7)
Supplement: Supplementary file 1 — Supplementary Figures [file 41416_2025_3170_MOESM1_ESM.docx]

**
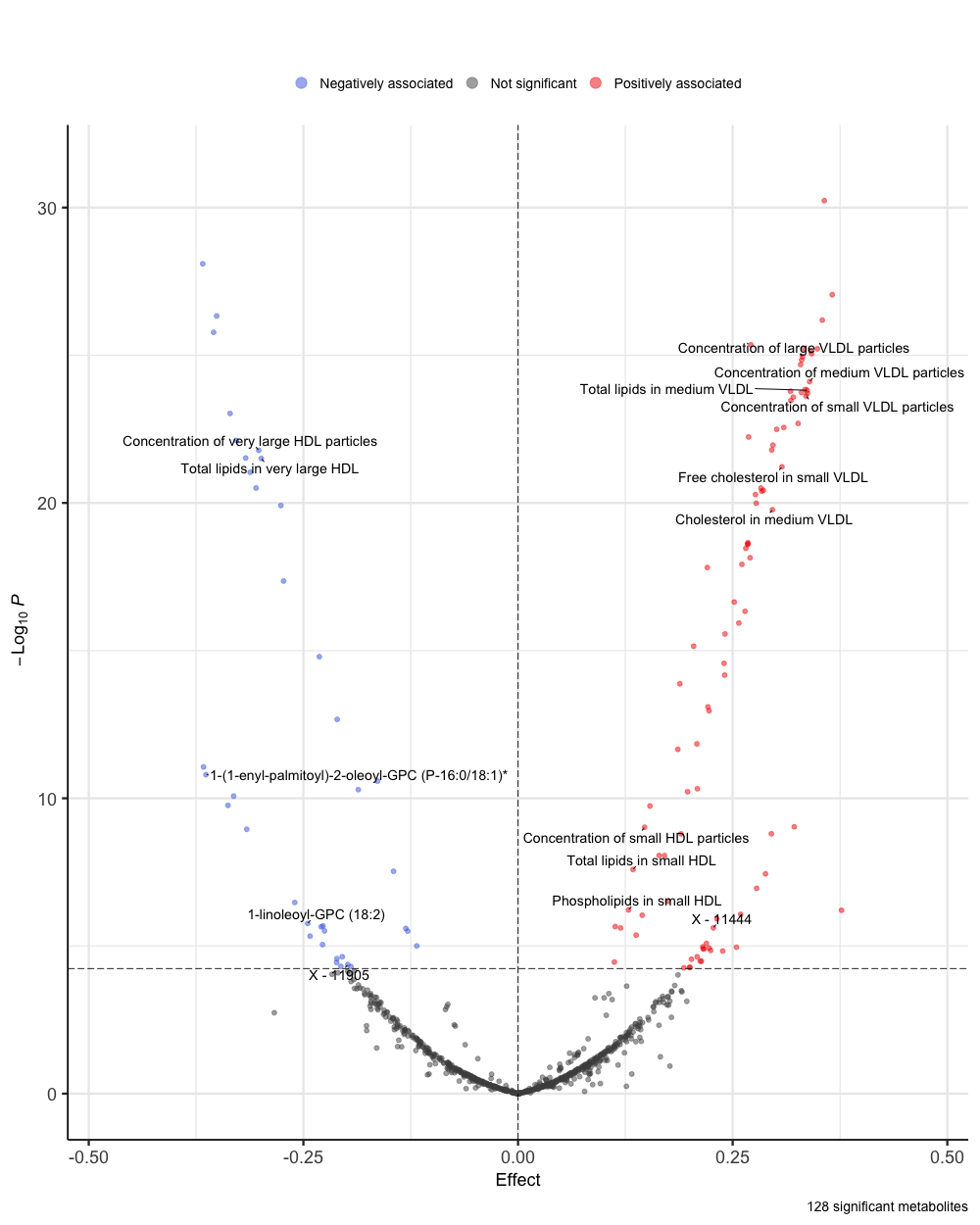
Supplementary Figure 1: Volcano plot of potentially causal associations between WHR and plasma metabolites found using MR.**

The dashed horizontal line indicates the Bonferroni-corrected significance threshold (*P =* 5.84 × 10^-5^) and all metabolites significantly associated with WHR are coloured. Metabolites that are negatively associated with WHR are coloured blue, whilst those positively associated with WHR are coloured red. The dashed vertical line indicates a null effect. Solid lines are used to connect labels to data points. The total number of significant metabolites is shown in the bottom-right corner. Created using the EnhancedVolcano (v1.20.0) R package.

**
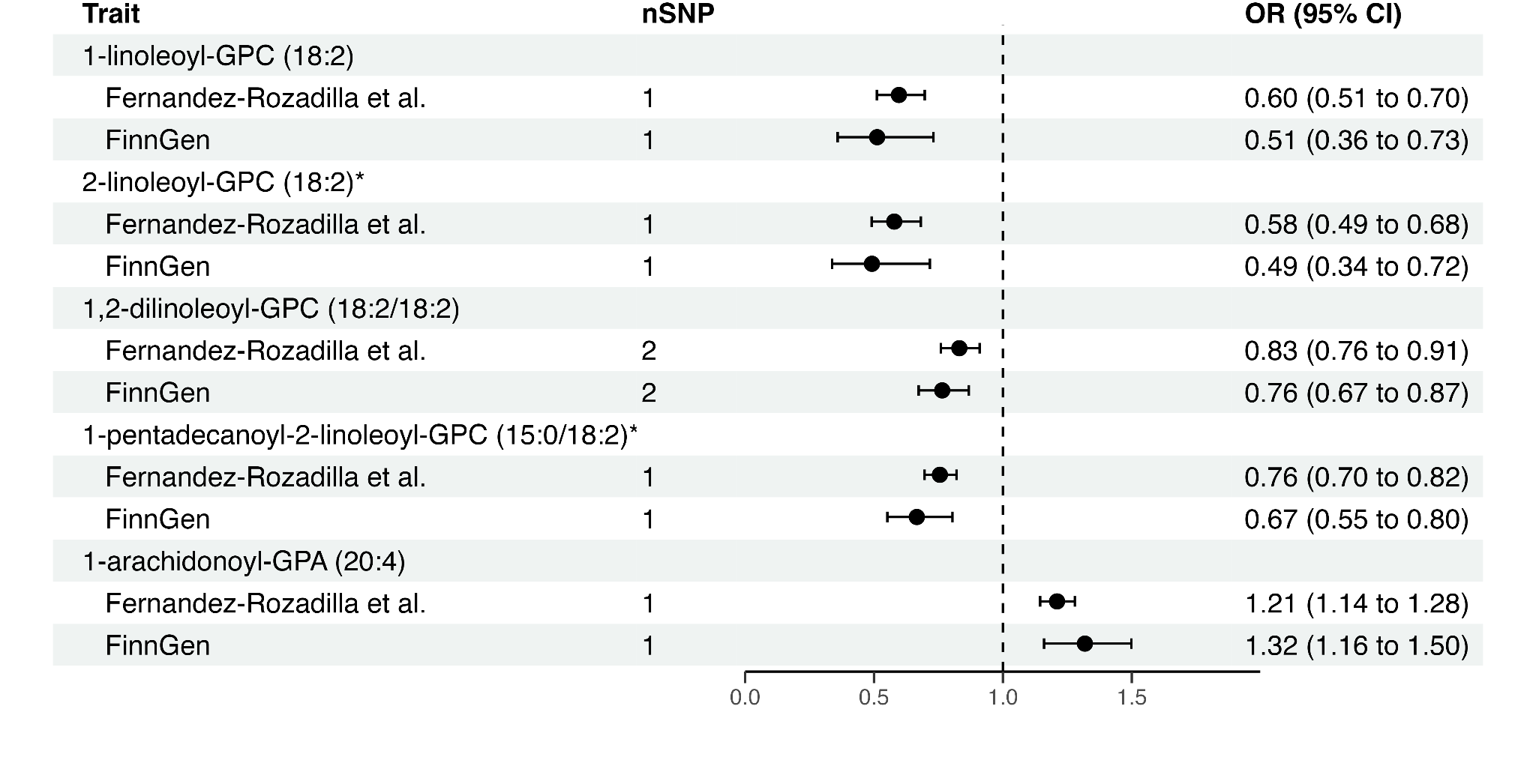
Supplementary Figure 2: Replication of obesity-driven metabolites associated with CRC risk.**

Each metabolite is shown with two data points: the first is the CRC GWAS used for discovery (Fernandez-Rozadilla et al.) and the second is the replication CRC GWAS (FinnGen). The vertical dashed line indicates a null effect. All associations are replicated in the FinnGen data. nSNP = number of SNPs. Created using the forester (v0.3.0) R package.

**
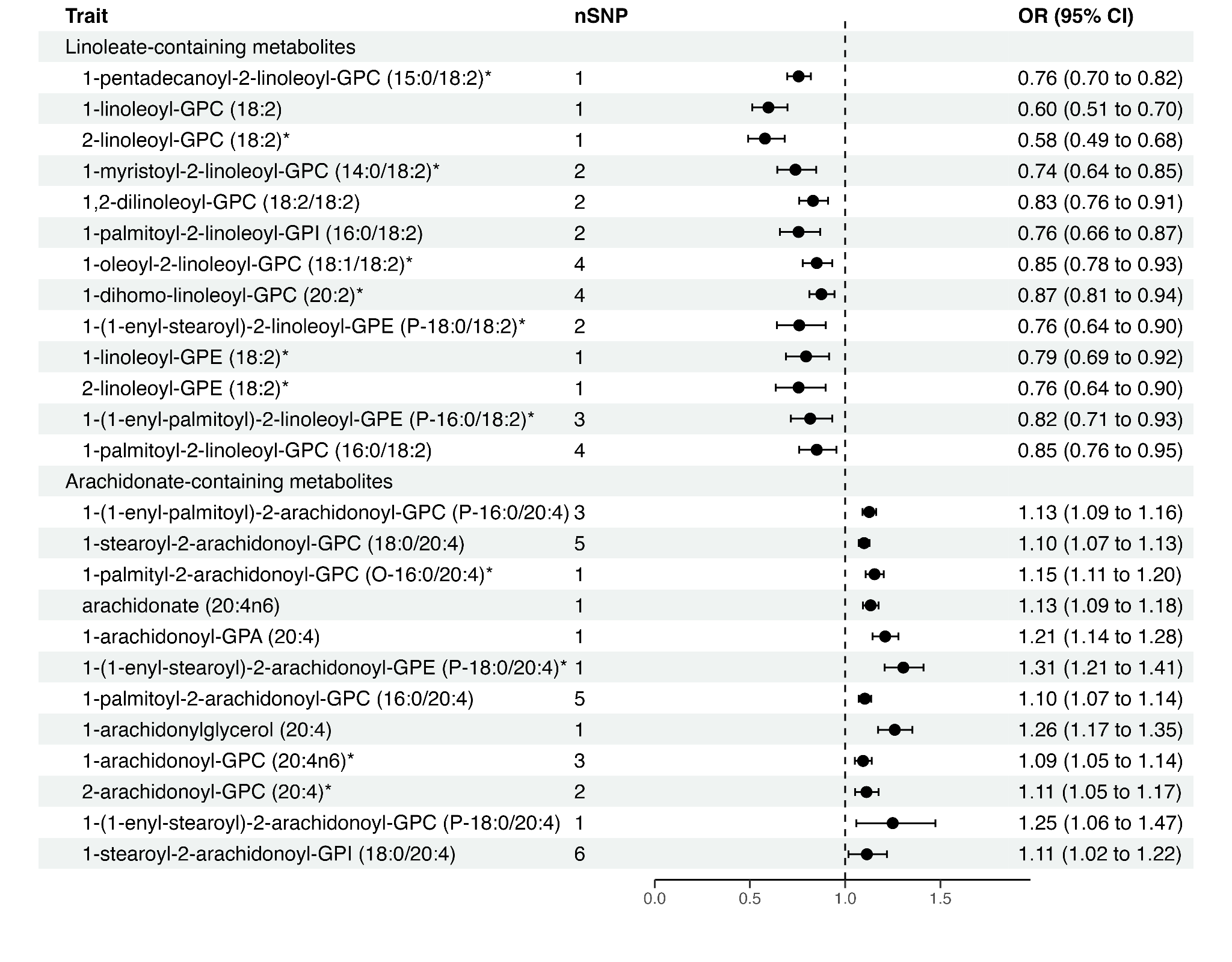
Supplementary Figure 3: Forest plot of linoleate- and arachidonate-containing metabolites associated with CRC risk.**

The names of the metabolites are given in the left column, stratified by whether they are precursors of linoleate or arachidonate. The vertical dashed line indicates a null effect. The error bars show 95% confidence intervals around the estimated odds ratio. nSNP = number of SNPs. Created using the forester (v0.3.0) R package.


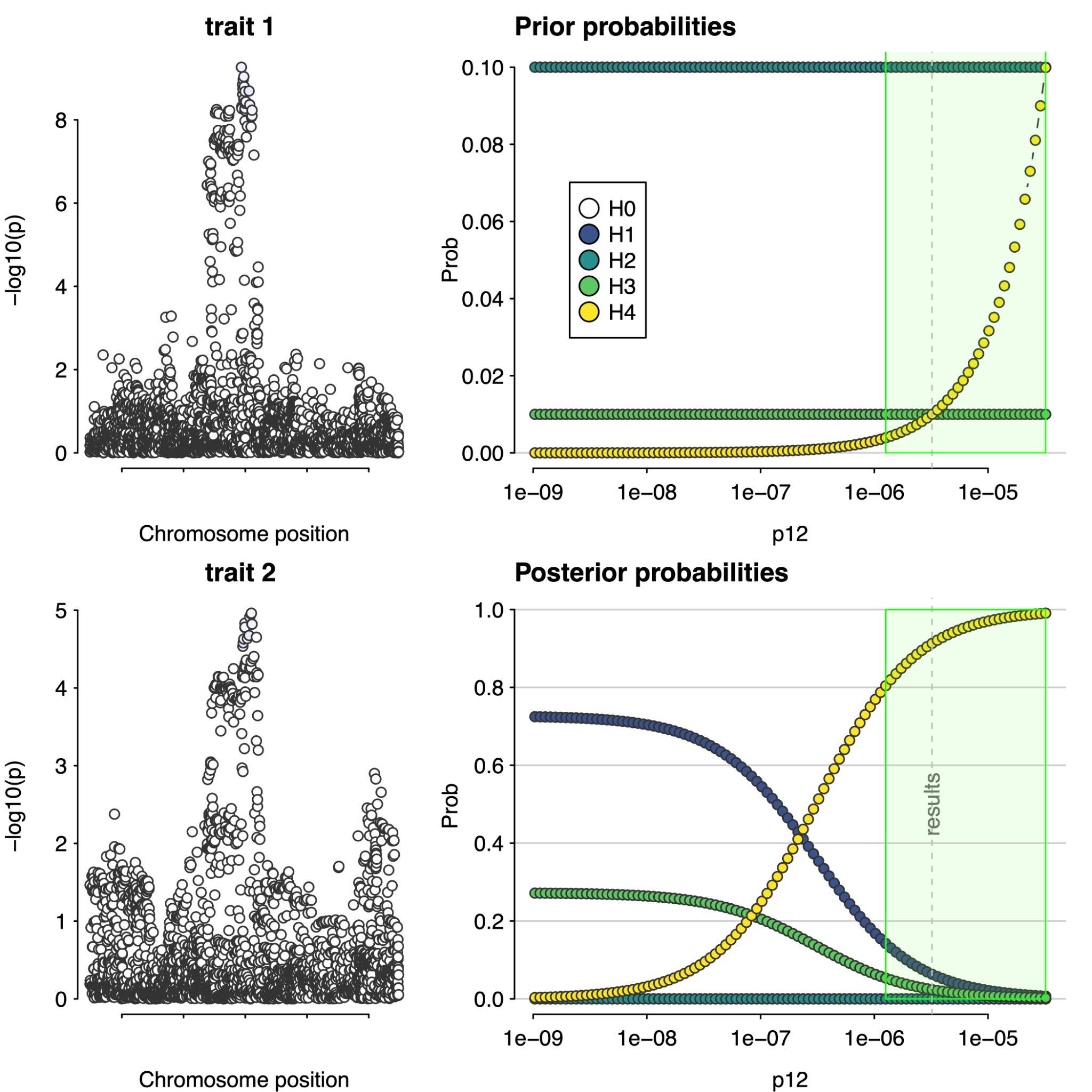


**Supplementary Figure 4: Colocalisation sensitivity analysis of 1-oleoyl-GPC and breast cancer.**

The left plots show the negative log-transformed *P*-values of every SNP within the 500 kb locus. The right plots show the prior and posterior probabilities of each of the five possible hypotheses for a given p12. The green box shows the range of p12 for which the colocalisation threshold (posterior probability of H4 > 0.8) is reached. The dashed line indicates the value of p12 chosen by the method described within the paper. Trait 1 is the metabolite exposure and trait 2 is the cancer outcome. H0-4 refer to the five possible hypotheses: H0, neither trait has a genetic association in the region; H1, only the exposure has a genetic association in the region; H2, only the outcome has a genetic association in the region; H3, both traits are associated, but with different causal variants; H4, the exposure and outcome are associated and share a single causal variant. Prob = probability. p12 is the prior probability that a given SNP within the locus is associated with both traits.


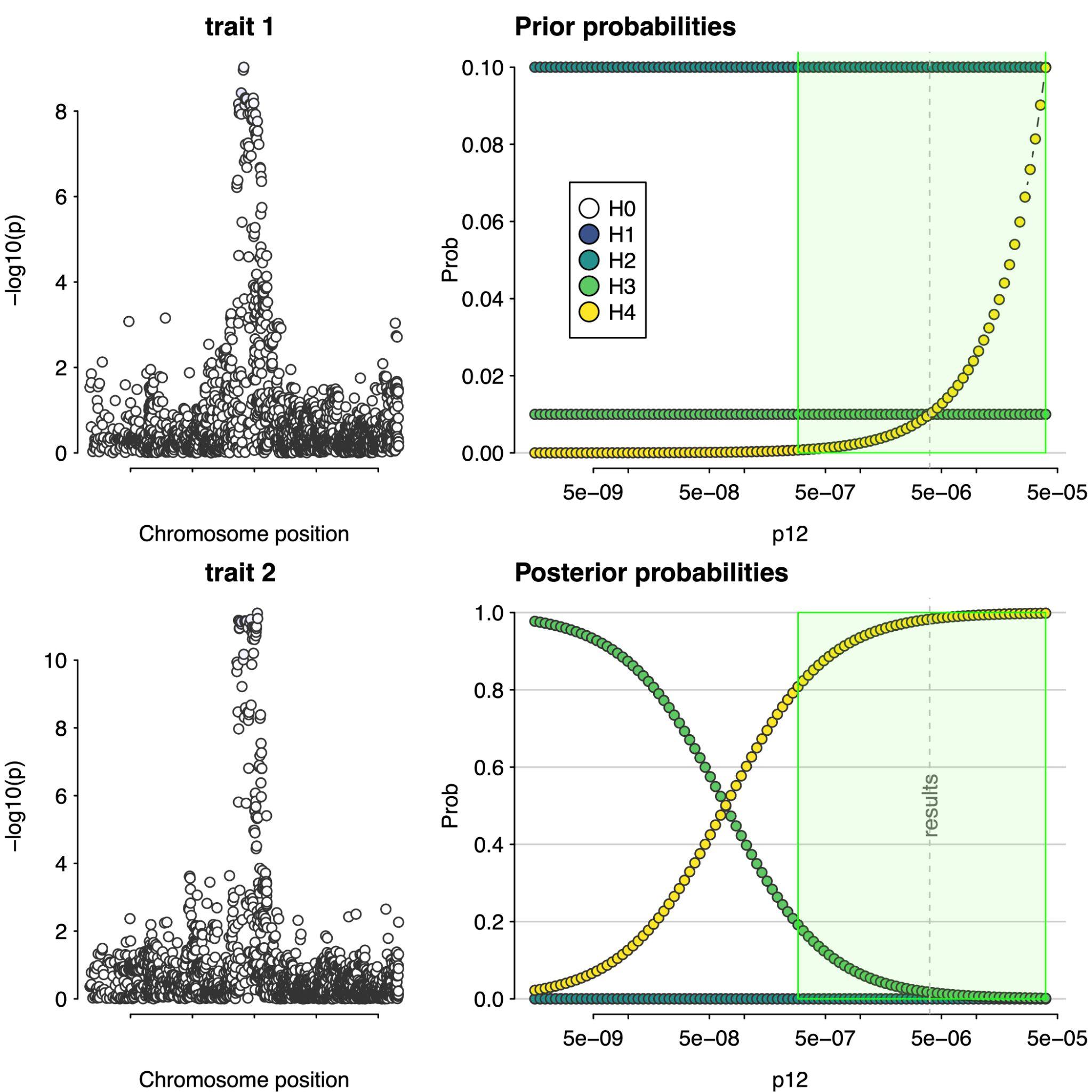


**Supplementary Figure 5: Colocalisation sensitivity analysis of 1-linoleoyl-GPC and colorectal cancer.**

The left plots show the negative log-transformed *P*-values of every SNP within the 500 kb locus. The right plots show the prior and posterior probabilities of each of the five possible hypotheses for a given p12. The green box shows the range of p12 for which the colocalisation threshold (posterior probability of H4 > 0.8) is reached. The dashed line indicates the value of p12 chosen by the method described within the paper. Trait 1 is the metabolite exposure and trait 2 is the cancer outcome. H0-4 refer to the five possible hypotheses: H0, neither trait has a genetic association in the region; H1, only the exposure has a genetic association in the region; H2, only the outcome has a genetic association in the region; H3, both traits are associated, but with different causal variants; H4, the exposure and outcome are associated and share a single causal variant. Prob = probability. p12 is the prior probability that a given SNP within the locus is associated with both traits.


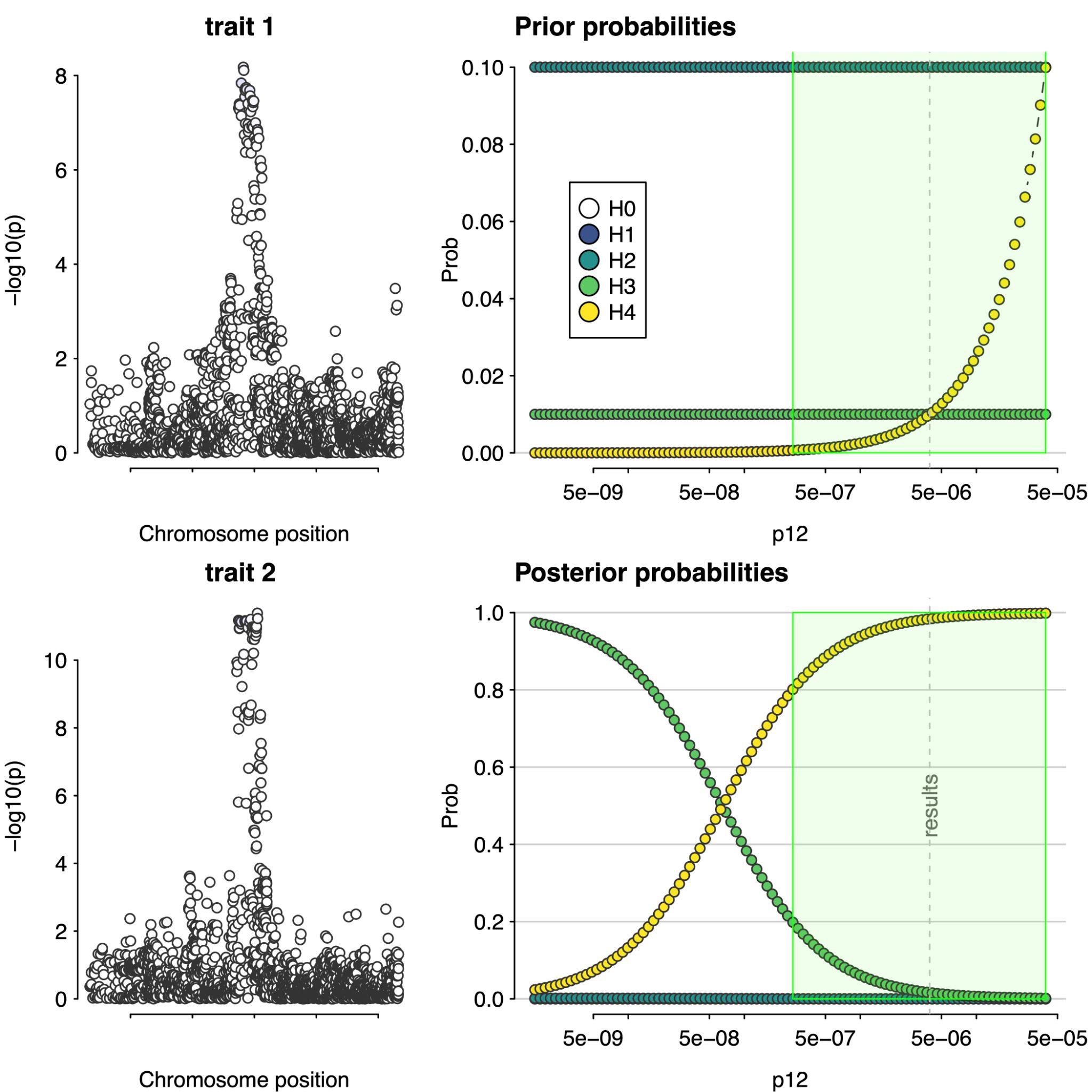


**Supplementary Figure 6: Colocalisation sensitivity analysis of 2-linoleoyl-GPC and colorectal cancer.**

The left plots show the negative log-transformed *P*-values of every SNP within the 500 kb locus. The right plots show the prior and posterior probabilities of each of the five possible hypotheses for a given p12. The green box shows the range of p12 for which the colocalisation threshold (posterior probability of H4 > 0.8) is reached. The dashed line indicates the value of p12 chosen by the method described within the paper. Trait 1 is the metabolite exposure and trait 2 is the cancer outcome. H0-4 refer to the five possible hypotheses: H0, neither trait has a genetic association in the region; H1, only the exposure has a genetic association in the region; H2, only the outcome has a genetic association in the region; H3, both traits are associated, but with different causal variants; H4, the exposure and outcome are associated and share a single causal variant. Prob = probability. p12 is the prior probability that a given SNP within the locus is associated with both traits.


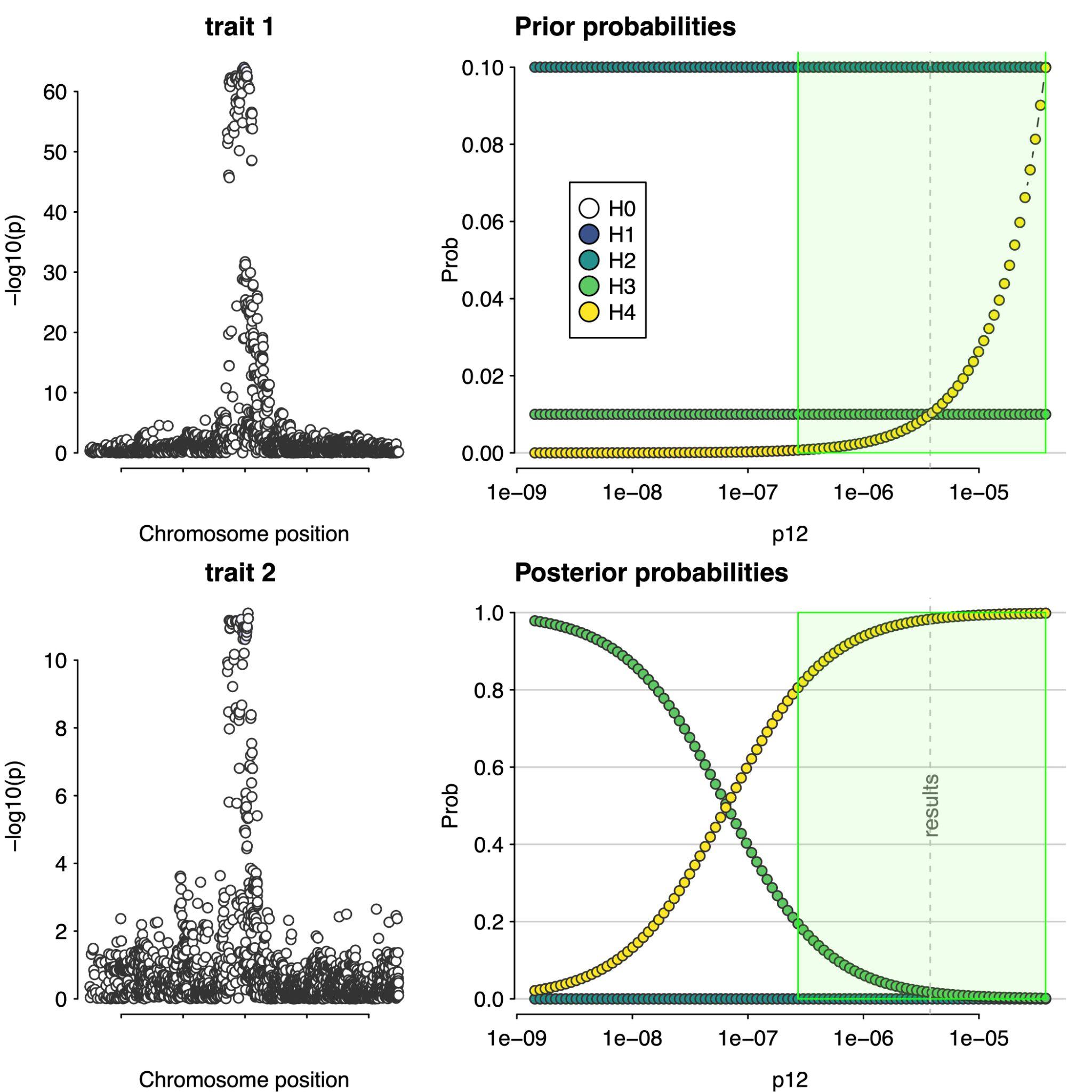


**Supplementary Figure 7: Colocalisation sensitivity analysis of 1-arachidonoyl-GPA and colorectal cancer.**

The left plots show the negative log-transformed *P*-values of every SNP within the 500 kb locus. The right plots show the prior and posterior probabilities of each of the five possible hypotheses for a given p12. The green box shows the range of p12 for which the colocalisation threshold (posterior probability of H4 > 0.8) is reached. The dashed line indicates the value of p12 chosen by the method described within the paper. Trait 1 is the metabolite exposure and trait 2 is the cancer outcome. H0-4 refer to the five possible hypotheses: H0, neither trait has a genetic association in the region; H1, only the exposure has a genetic association in the region; H2, only the outcome has a genetic association in the region; H3, both traits are associated, but with different causal variants; H4, the exposure and outcome are associated and share a single causal variant. Prob = probability. p12 is the prior probability that a given SNP within the locus is associated with both traits.


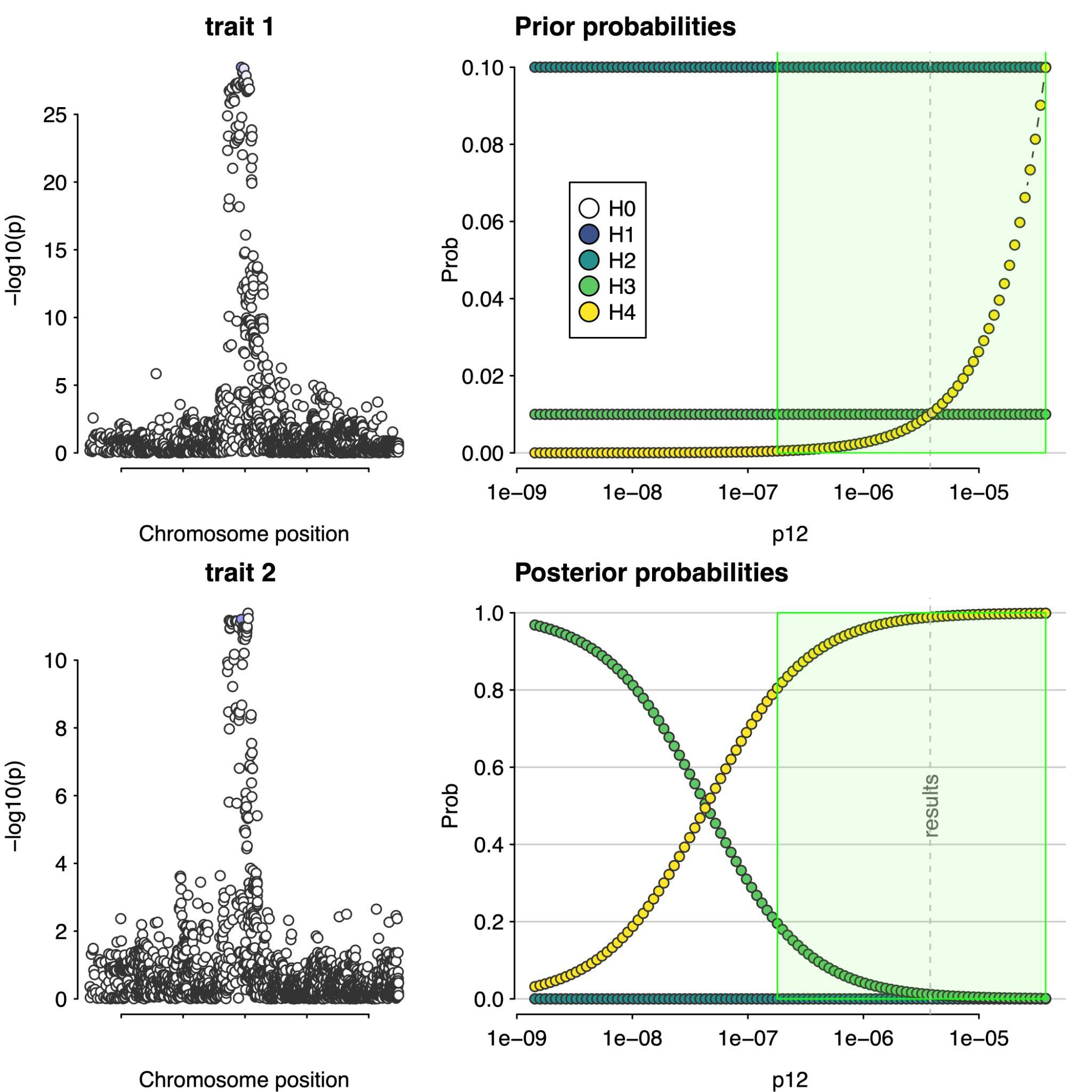


**Supplementary Figure 8: Colocalisation sensitivity analysis of 1-pentadecanoyl-2-linoleoyl-GPC and colorectal cancer.**

The left plots show the negative log-transformed *P*-values of every SNP within the 500 kb locus. The right plots show the prior and posterior probabilities of each of the five possible hypotheses for a given p12. The green box shows the range of p12 for which the colocalisation threshold (posterior probability of H4 > 0.8) is reached. The dashed line indicates the value of p12 chosen by the method described within the paper. Trait 1 is the metabolite exposure and trait 2 is the cancer outcome. H0-4 refer to the five possible hypotheses: H0, neither trait has a genetic association in the region; H1, only the exposure has a genetic association in the region; H2, only the outcome has a genetic association in the region; H3, both traits are associated, but with different causal variants; H4, the exposure and outcome are associated and share a single causal variant. Prob = probability. p12 is the prior probability that a given SNP within the locus is associated with both traits.


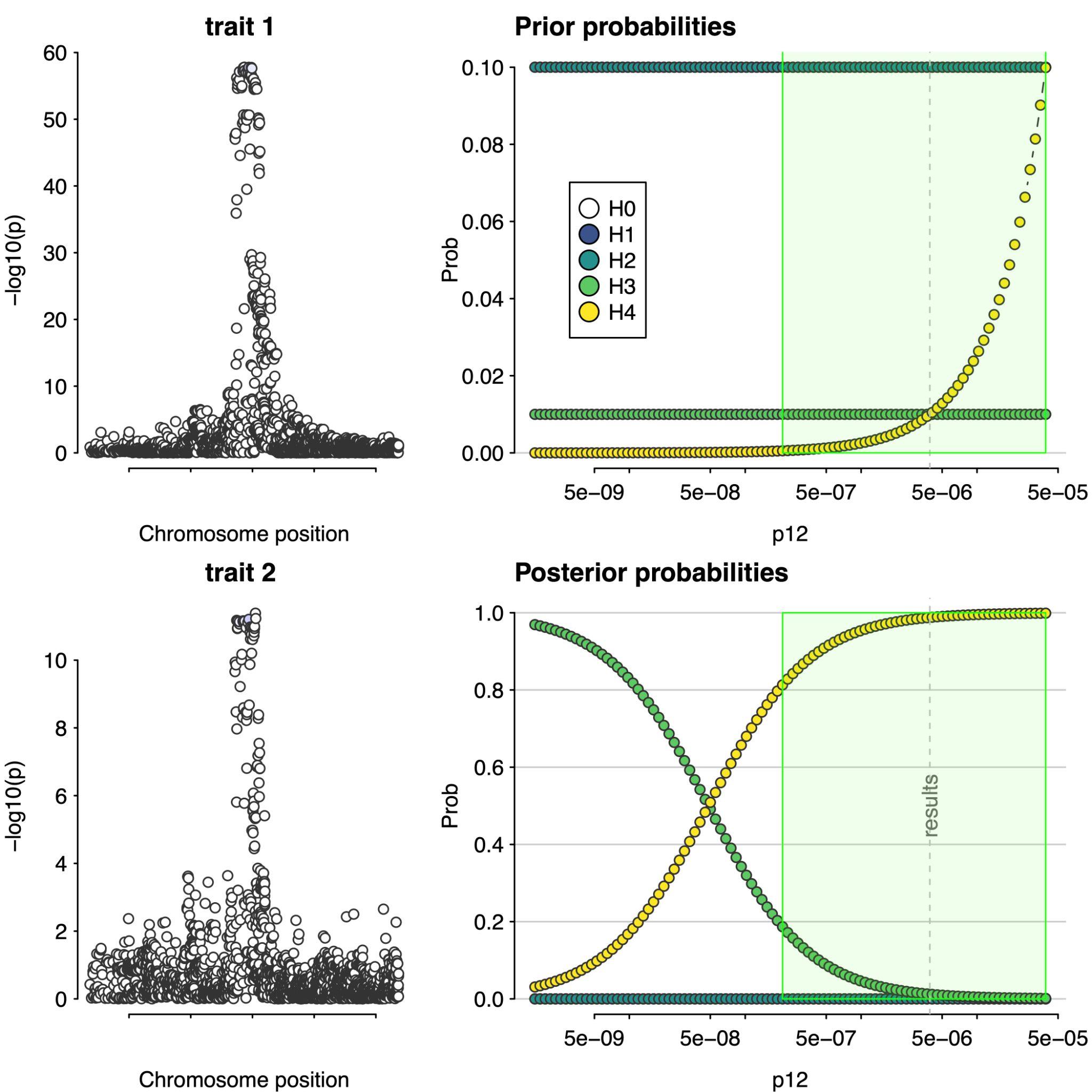


**Supplementary Figure 9: Colocalisation sensitivity analysis of 1,2-dilinoleoyl-GPC and colorectal cancer.**

The left plots show the negative log-transformed *P*-values of every SNP within the 500 kb locus. The right plots show the prior and posterior probabilities of each of the five possible hypotheses for a given p12. The green box shows the range of p12 for which the colocalisation threshold (posterior probability of H4 > 0.8) is reached. The dashed line indicates the value of p12 chosen by the method described within the paper. Trait 1 is the metabolite exposure and trait 2 is the cancer outcome. H0-4 refer to the five possible hypotheses: H0, neither trait has a genetic association in the region; H1, only the exposure has a genetic association in the region; H2, only the outcome has a genetic association in the region; H3, both traits are associated, but with different causal variants; H4, the exposure and outcome are associated and share a single causal variant. Prob = probability. p12 is the prior probability that a given SNP within the locus is associated with both traits.


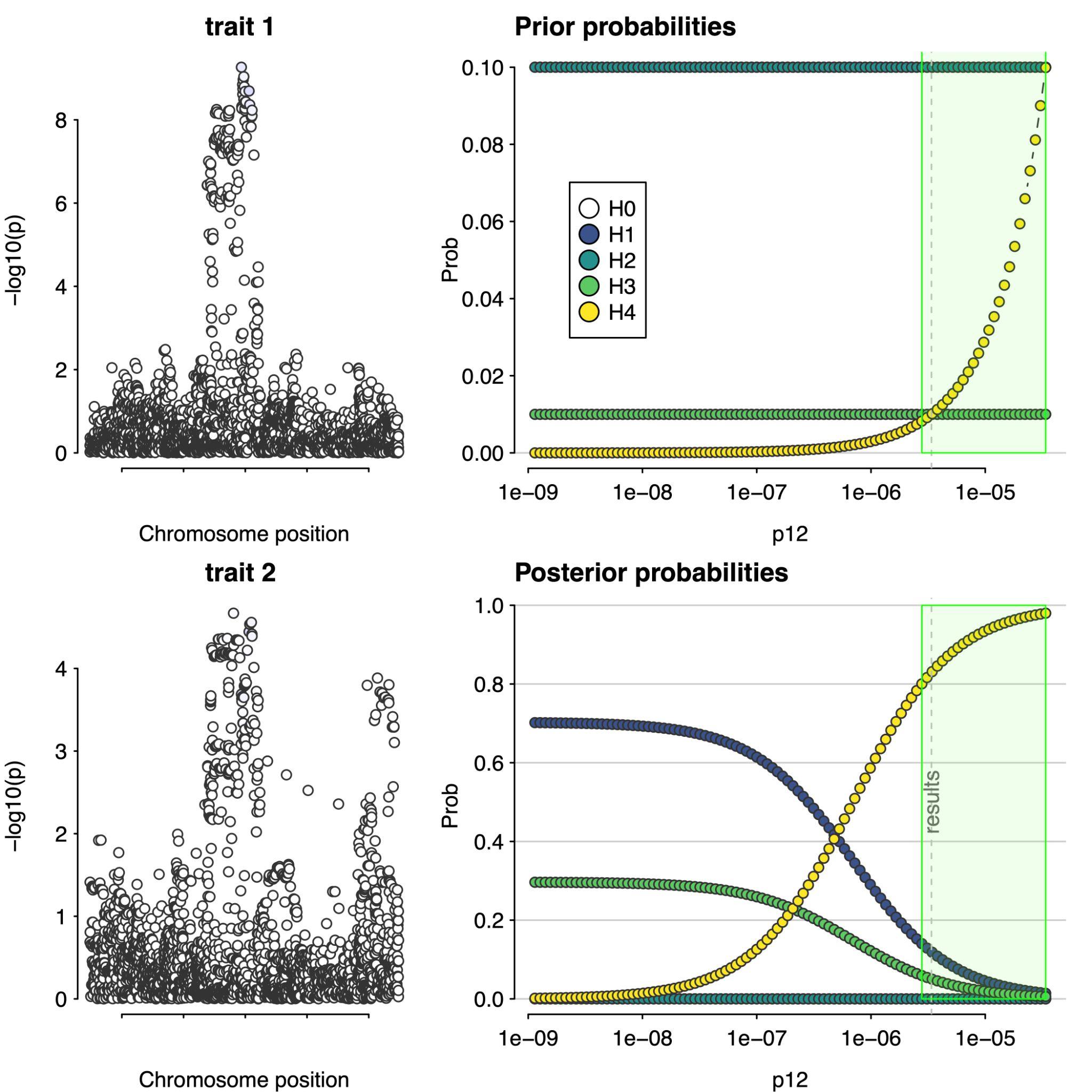


**Supplementary Figure 10: Colocalisation sensitivity analysis of 1-oleoyl-GPC and luminal-A breast cancer.**

The left plots show the negative log-transformed *P*-values of every SNP within the 500 kb locus. The right plots show the prior and posterior probabilities of each of the five possible hypotheses for a given p12. The green box shows the range of p12 for which the colocalisation threshold (posterior probability of H4 > 0.8) is reached. The dashed line indicates the value of p12 chosen by the method described within the paper. Trait 1 is the metabolite exposure and trait 2 is the cancer outcome. H0-4 refer to the five possible hypotheses: H0, neither trait has a genetic association in the region; H1, only the exposure has a genetic association in the region; H2, only the outcome has a genetic association in the region; H3, both traits are associated, but with different causal variants; H4, the exposure and outcome are associated and share a single causal variant. Prob = probability. p12 is the prior probability that a given SNP within the locus is associated with both traits.


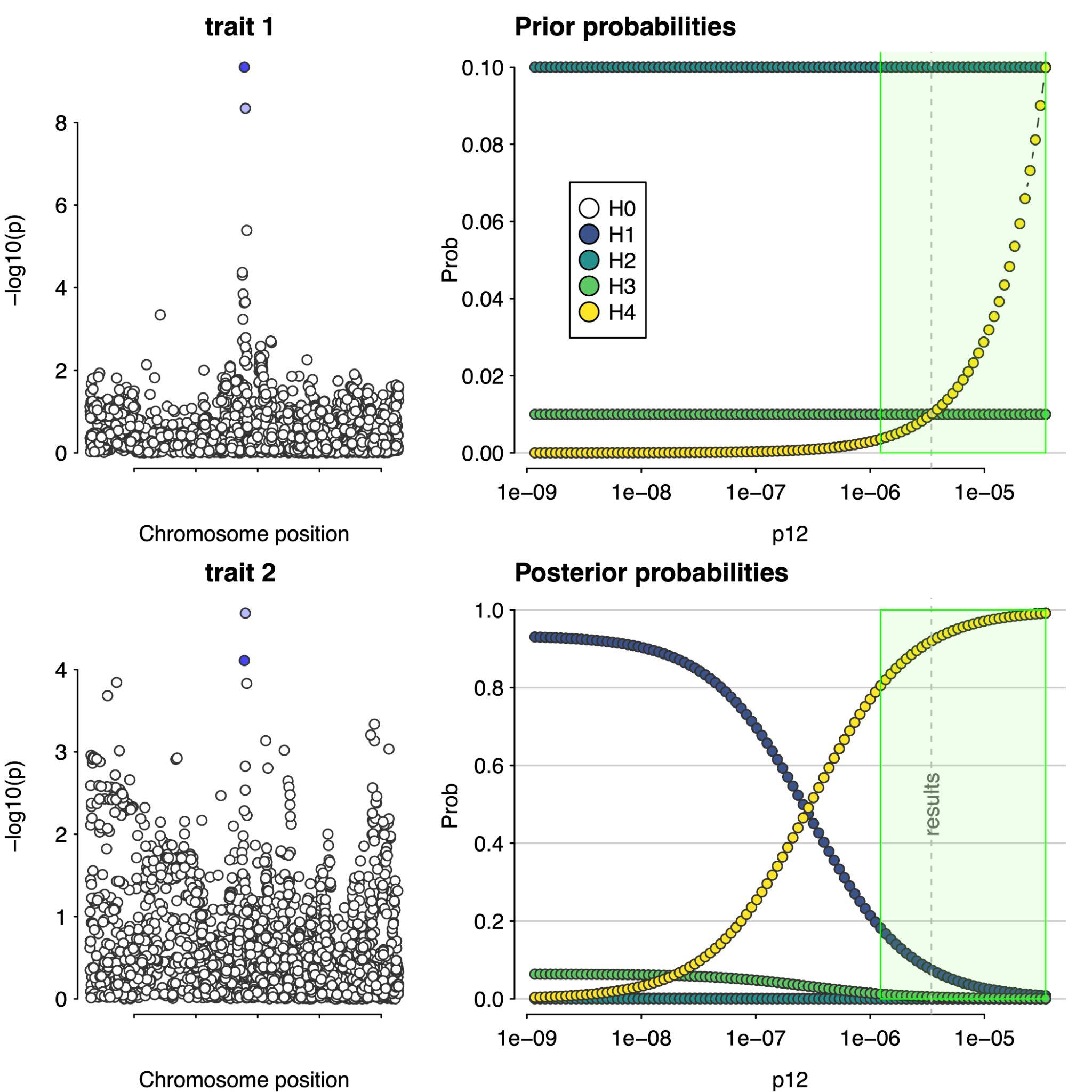


**Supplementary Figure 11: Colocalisation sensitivity analysis of concentration of medium VLDL particles and luminal-A breast cancer.**

The left plots show the negative log-transformed *P*-values of every SNP within the 500 kb locus. The right plots show the prior and posterior probabilities of each of the five possible hypotheses for a given p12. The green box shows the range of p12 for which the colocalisation threshold (posterior probability of H4 > 0.8) is reached. The dashed line indicates the value of p12 chosen by the method described within the paper. Trait 1 is the metabolite exposure and trait 2 is the cancer outcome. H0-4 refer to the five possible hypotheses: H0, neither trait has a genetic association in the region; H1, only the exposure has a genetic association in the region; H2, only the outcome has a genetic association in the region; H3, both traits are associated, but with different causal variants; H4, the exposure and outcome are associated and share a single causal variant. Prob = probability. p12 is the prior probability that a given SNP within the locus is associated with both traits.


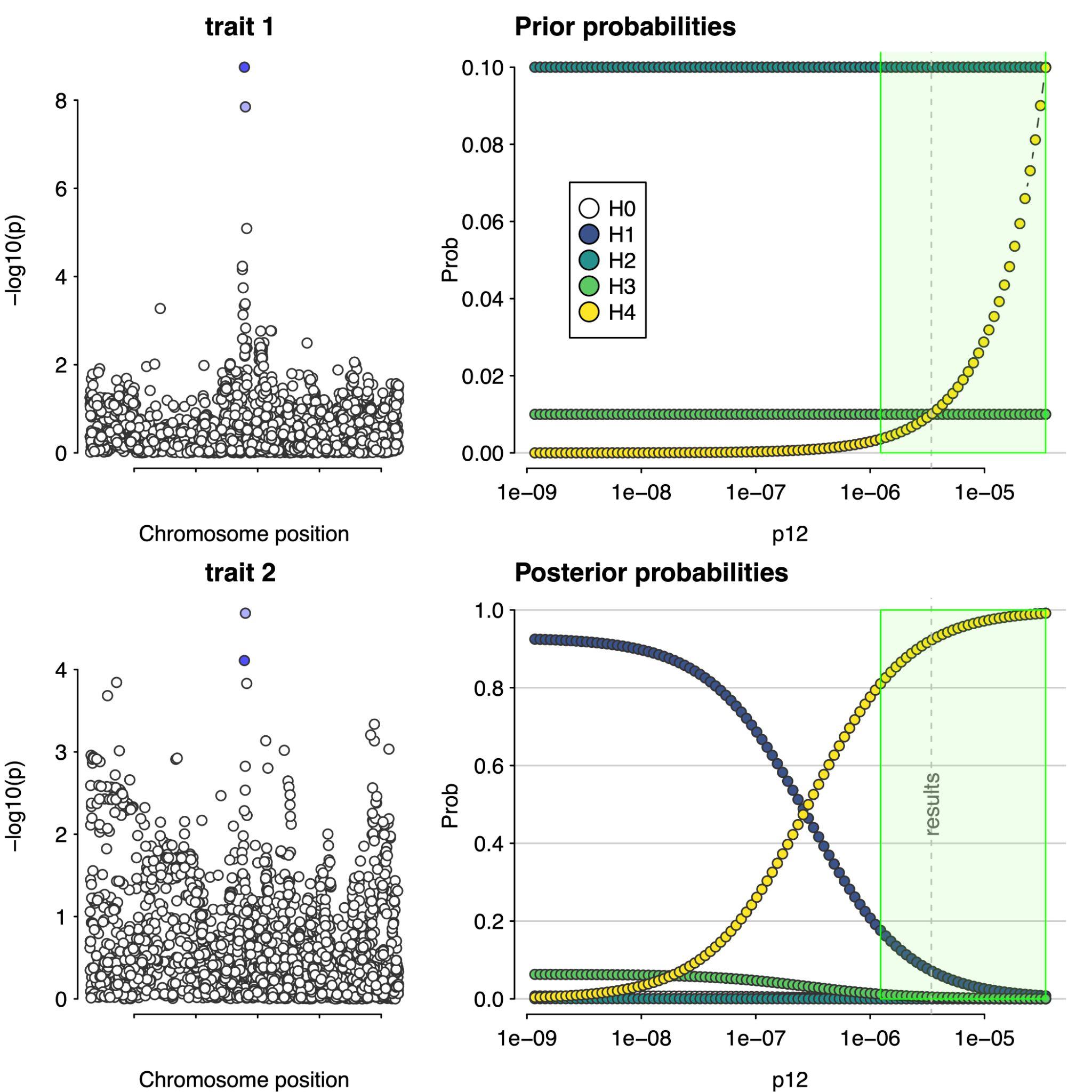


**Supplementary Figure 12: Colocalisation sensitivity analysis of phospholipids in medium VLDL and luminal-A breast cancer.**

The left plots show the negative log-transformed *P*-values of every SNP within the 500 kb locus. The right plots show the prior and posterior probabilities of each of the five possible hypotheses for a given p12. The green box shows the range of p12 for which the colocalisation threshold (posterior probability of H4 > 0.8) is reached. The dashed line indicates the value of p12 chosen by the method described within the paper. Trait 1 is the metabolite exposure and trait 2 is the cancer outcome. H0-4 refer to the five possible hypotheses: H0, neither trait has a genetic association in the region; H1, only the exposure has a genetic association in the region; H2, only the outcome has a genetic association in the region; H3, both traits are associated, but with different causal variants; H4, the exposure and outcome are associated and share a single causal variant. Prob = probability. p12 is the prior probability that a given SNP within the locus is associated with both traits.


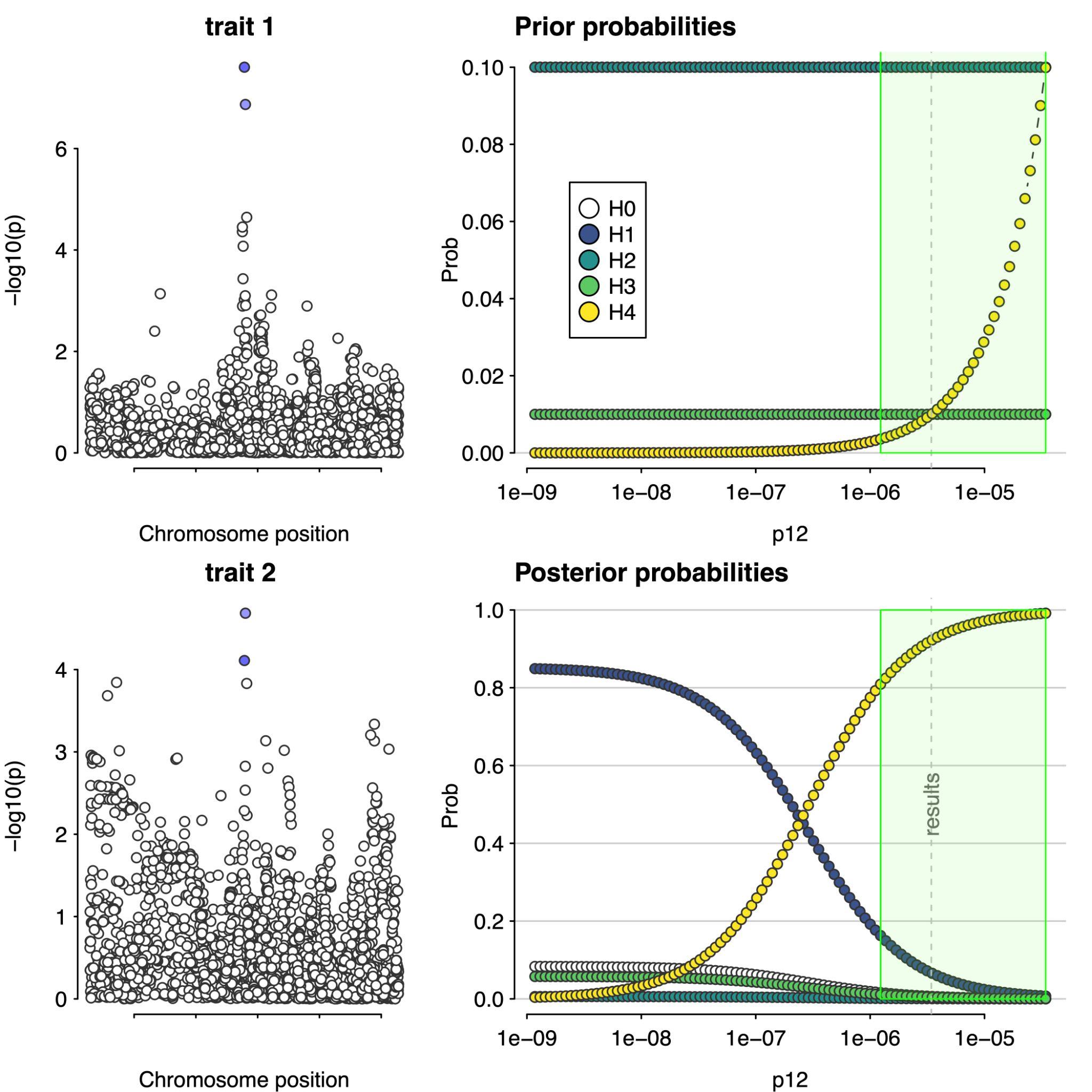


**Supplementary Figure 13: Colocalisation sensitivity analysis of concentration of small VLDL particles and luminal-A breast cancer.**

The left plots show the negative log-transformed *P*-values of every SNP within the 500 kb locus. The right plots show the prior and posterior probabilities of each of the five possible hypotheses for a given p12. The green box shows the range of p12 for which the colocalisation threshold (posterior probability of H4 > 0.8) is reached. The dashed line indicates the value of p12 chosen by the method described within the paper. Trait 1 is the metabolite exposure and trait 2 is the cancer outcome. H0-4 refer to the five possible hypotheses: H0, neither trait has a genetic association in the region; H1, only the exposure has a genetic association in the region; H2, only the outcome has a genetic association in the region; H3, both traits are associated, but with different causal variants; H4, the exposure and outcome are associated and share a single causal variant. Prob = probability. p12 is the prior probability that a given SNP within the locus is associated with both traits.


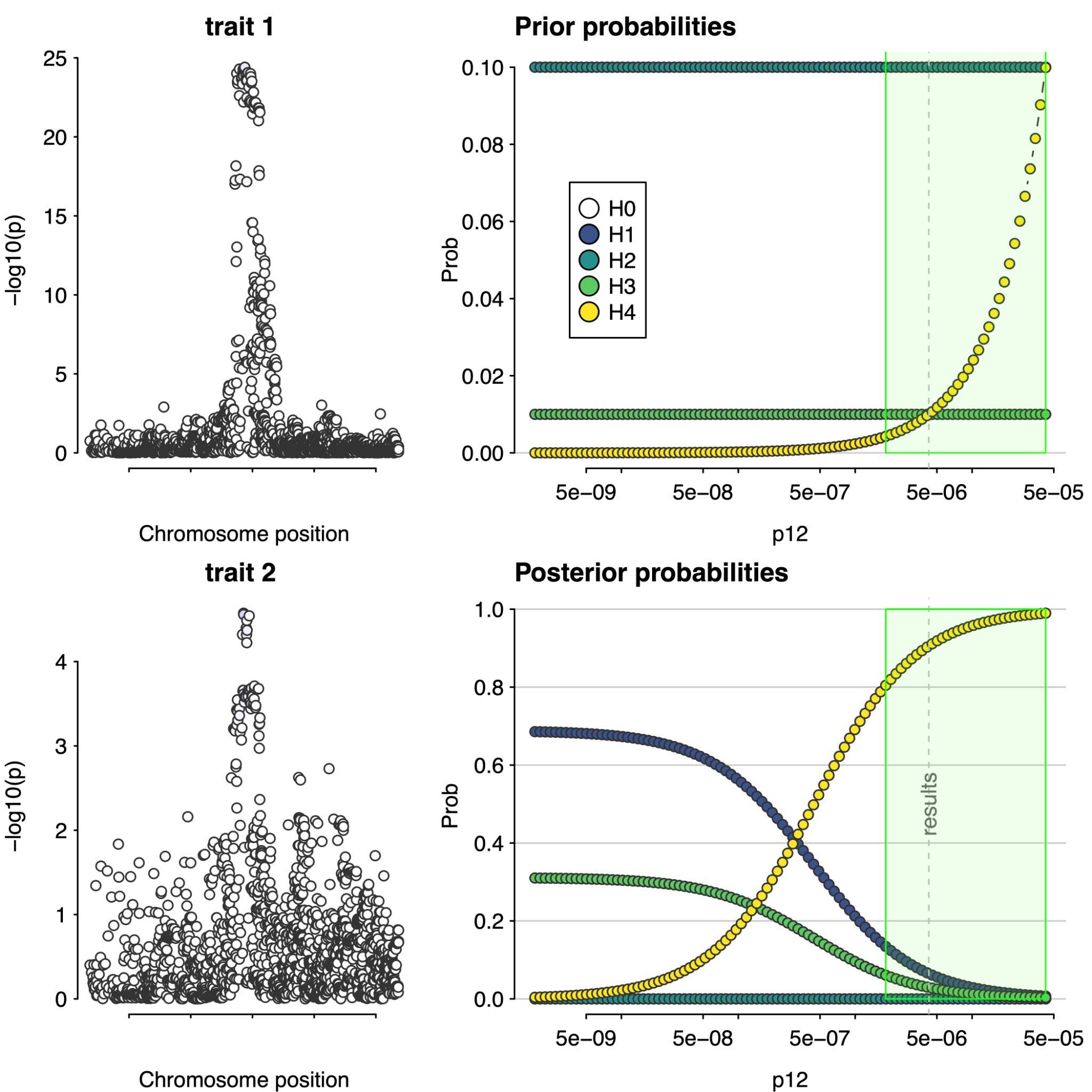


**Supplementary Figure 14: Colocalisation sensitivity analysis of 2-linoleoyl-GPE and rectal cancer.**

The left plots show the negative log-transformed *P*-values of every SNP within the 500 kb locus. The right plots show the prior and posterior probabilities of each of the five possible hypotheses for a given p12. The green box shows the range of p12 for which the colocalisation threshold (posterior probability of H4 > 0.8) is reached. The dashed line indicates the value of p12 chosen by the method described within the paper. Trait 1 is the metabolite exposure and trait 2 is the cancer outcome. H0-4 refer to the five possible hypotheses: H0, neither trait has a genetic association in the region; H1, only the exposure has a genetic association in the region; H2, only the outcome has a genetic association in the region; H3, both traits are associated, but with different causal variants; H4, the exposure and outcome are associated and share a single causal variant. Prob = probability. p12 is the prior probability that a given SNP within the locus is associated with both traits.


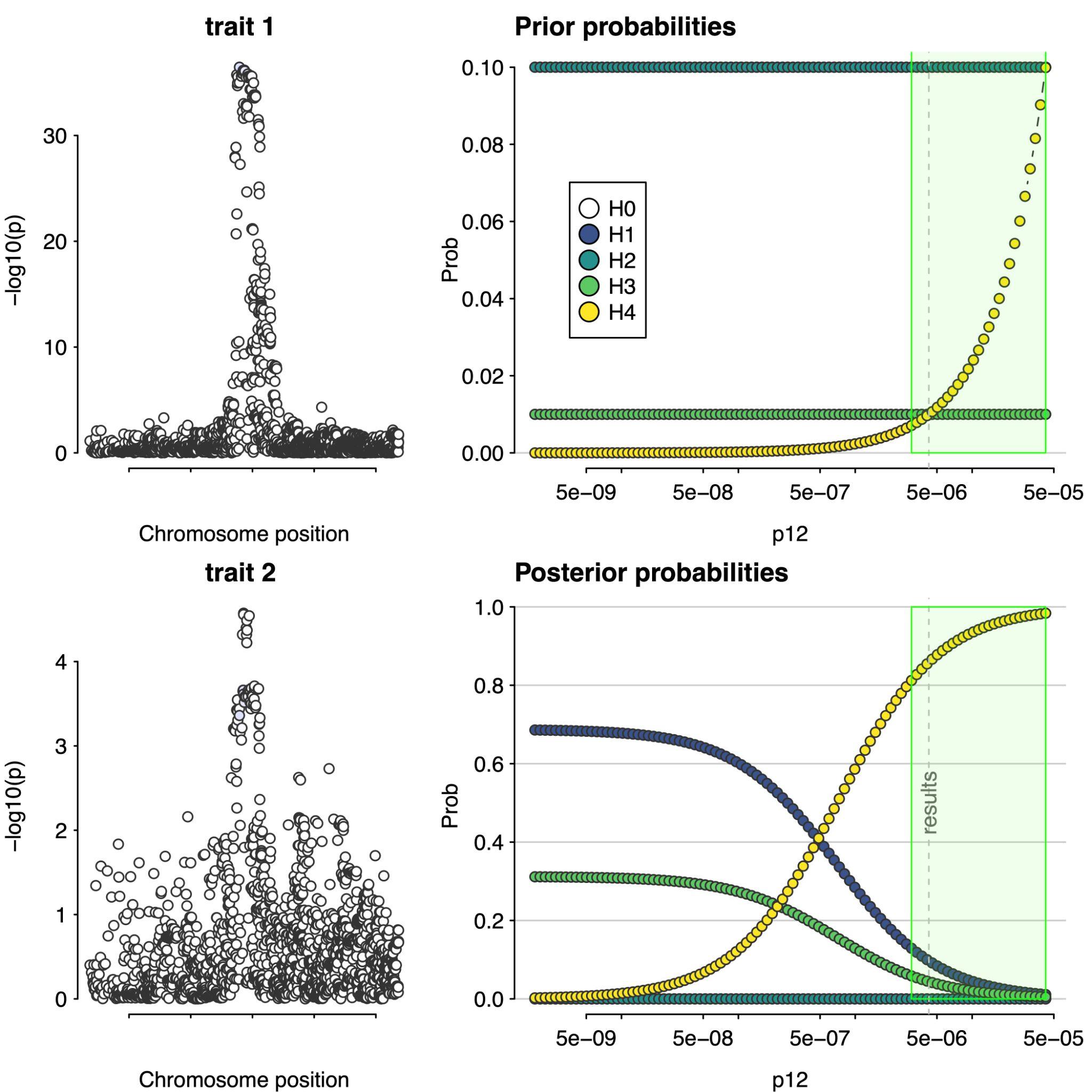


**Supplementary Figure 15: Colocalisation sensitivity analysis of 1-linoleoyl-GPE and rectal cancer.**

The left plots show the negative log-transformed *P*-values of every SNP within the 500 kb locus. The right plots show the prior and posterior probabilities of each of the five possible hypotheses for a given p12. The green box shows the range of p12 for which the colocalisation threshold (posterior probability of H4 > 0.8) is reached. The dashed line indicates the value of p12 chosen by the method described within the paper. Trait 1 is the metabolite exposure and trait 2 is the cancer outcome. H0-4 refer to the five possible hypotheses: H0, neither trait has a genetic association in the region; H1, only the exposure has a genetic association in the region; H2, only the outcome has a genetic association in the region; H3, both traits are associated, but with different causal variants; H4, the exposure and outcome are associated and share a single causal variant. Prob = probability. p12 is the prior probability that a given SNP within the locus is associated with both traits.


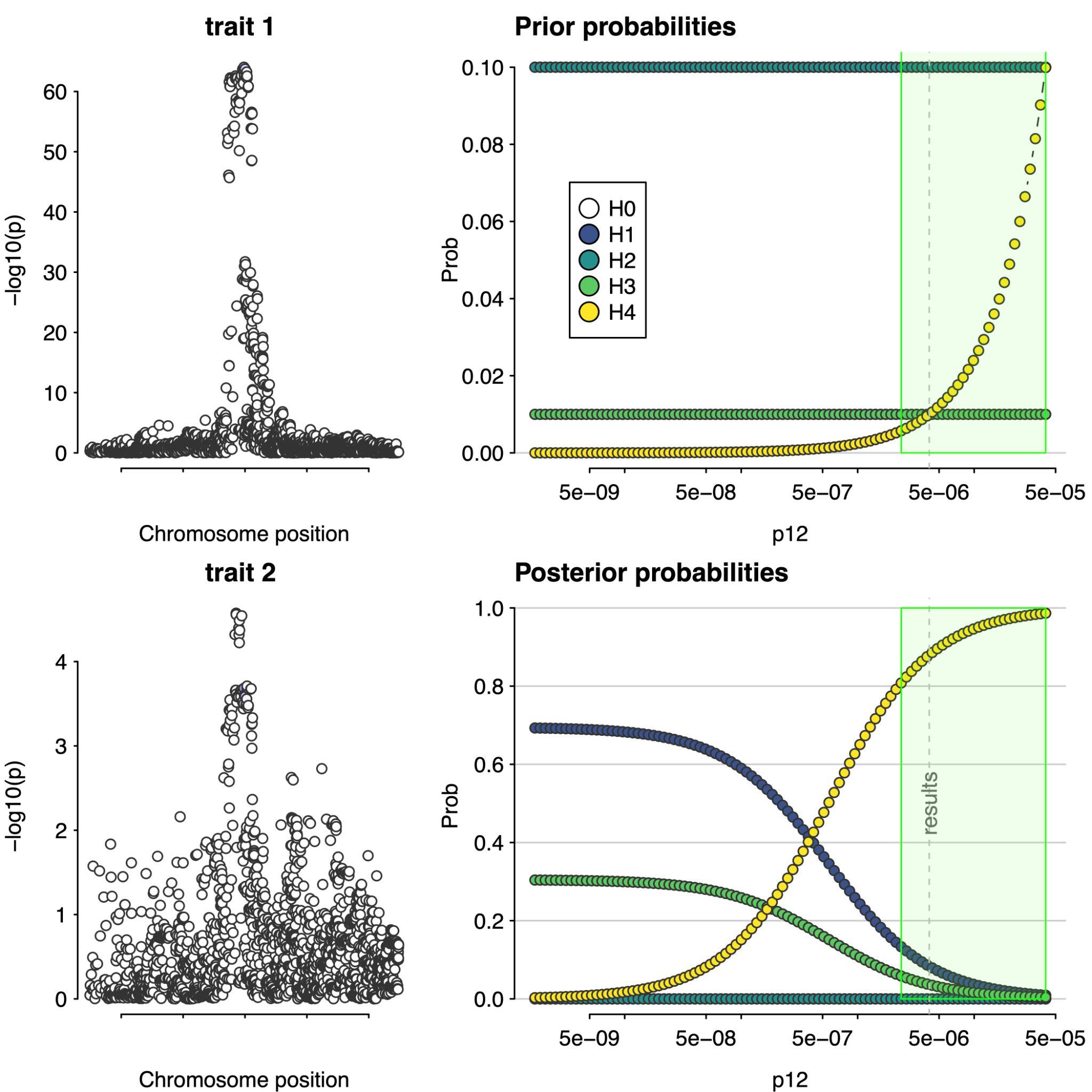


**Supplementary Figure 16: Colocalisation sensitivity analysis of 1-arachidonoyl-GPA and rectal cancer.**

The left plots show the negative log-transformed *P*-values of every SNP within the 500 kb locus. The right plots show the prior and posterior probabilities of each of the five possible hypotheses for a given p12. The green box shows the range of p12 for which the colocalisation threshold (posterior probability of H4 > 0.8) is reached. The dashed line indicates the value of p12 chosen by the method described within the paper. Trait 1 is the metabolite exposure and trait 2 is the cancer outcome. H0-4 refer to the five possible hypotheses: H0, neither trait has a genetic association in the region; H1, only the exposure has a genetic association in the region; H2, only the outcome has a genetic association in the region; H3, both traits are associated, but with different causal variants; H4, the exposure and outcome are associated and share a single causal variant. Prob = probability. p12 is the prior probability that a given SNP within the locus is associated with both traits.


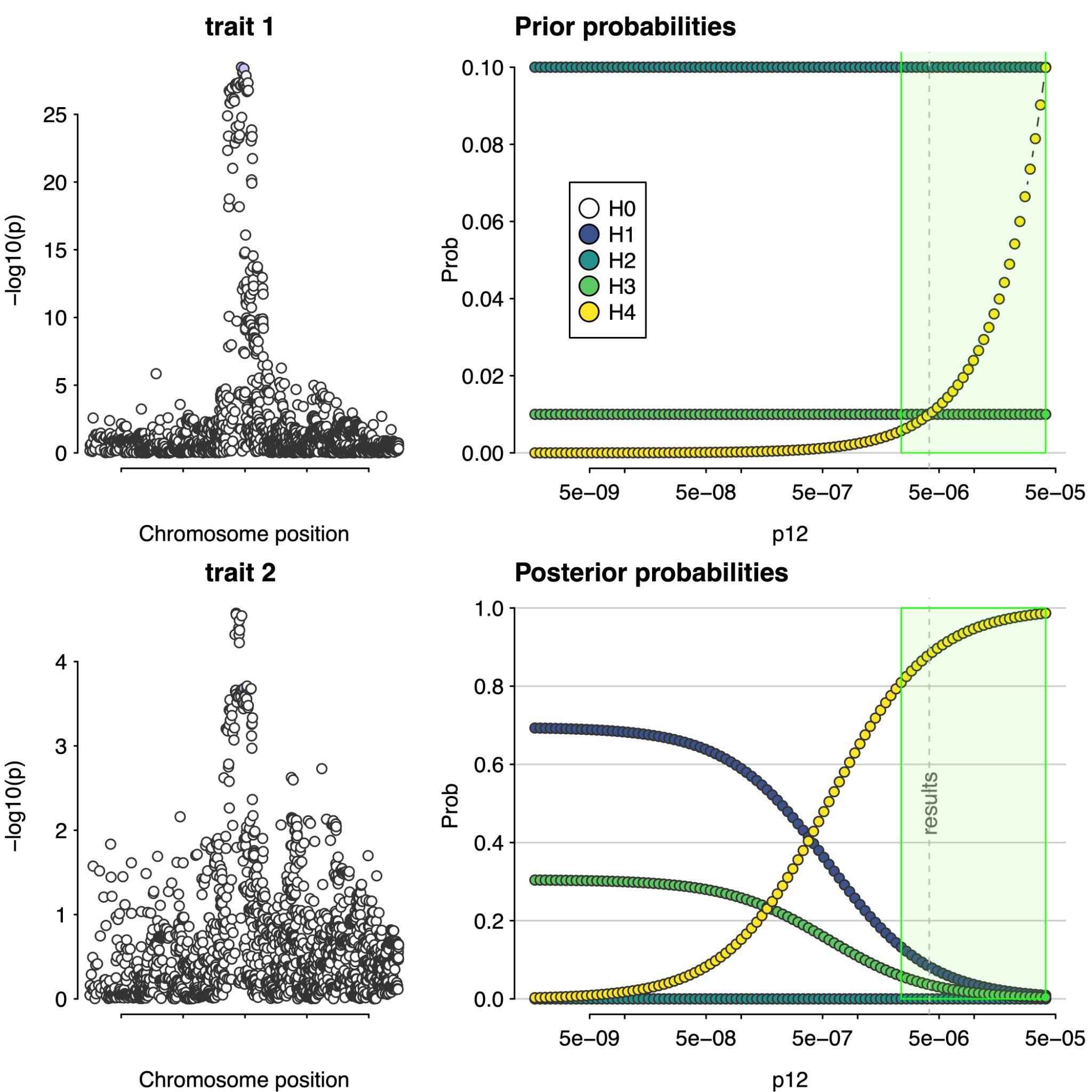


**Supplementary Figure 17: Colocalisation sensitivity analysis of 1-pentadecanoyl-2-linoleoyl-GPC and rectal cancer.**

The left plots show the negative log-transformed *P*-values of every SNP within the 500 kb locus. The right plots show the prior and posterior probabilities of each of the five possible hypotheses for a given p12. The green box shows the range of p12 for which the colocalisation threshold (posterior probability of H4 > 0.8) is reached. The dashed line indicates the value of p12 chosen by the method described within the paper. Trait 1 is the metabolite exposure and trait 2 is the cancer outcome. H0-4 refer to the five possible hypotheses: H0, neither trait has a genetic association in the region; H1, only the exposure has a genetic association in the region; H2, only the outcome has a genetic association in the region; H3, both traits are associated, but with different causal variants; H4, the exposure and outcome are associated and share a single causal variant. Prob = probability. p12 is the prior probability that a given SNP within the locus is associated with both traits.


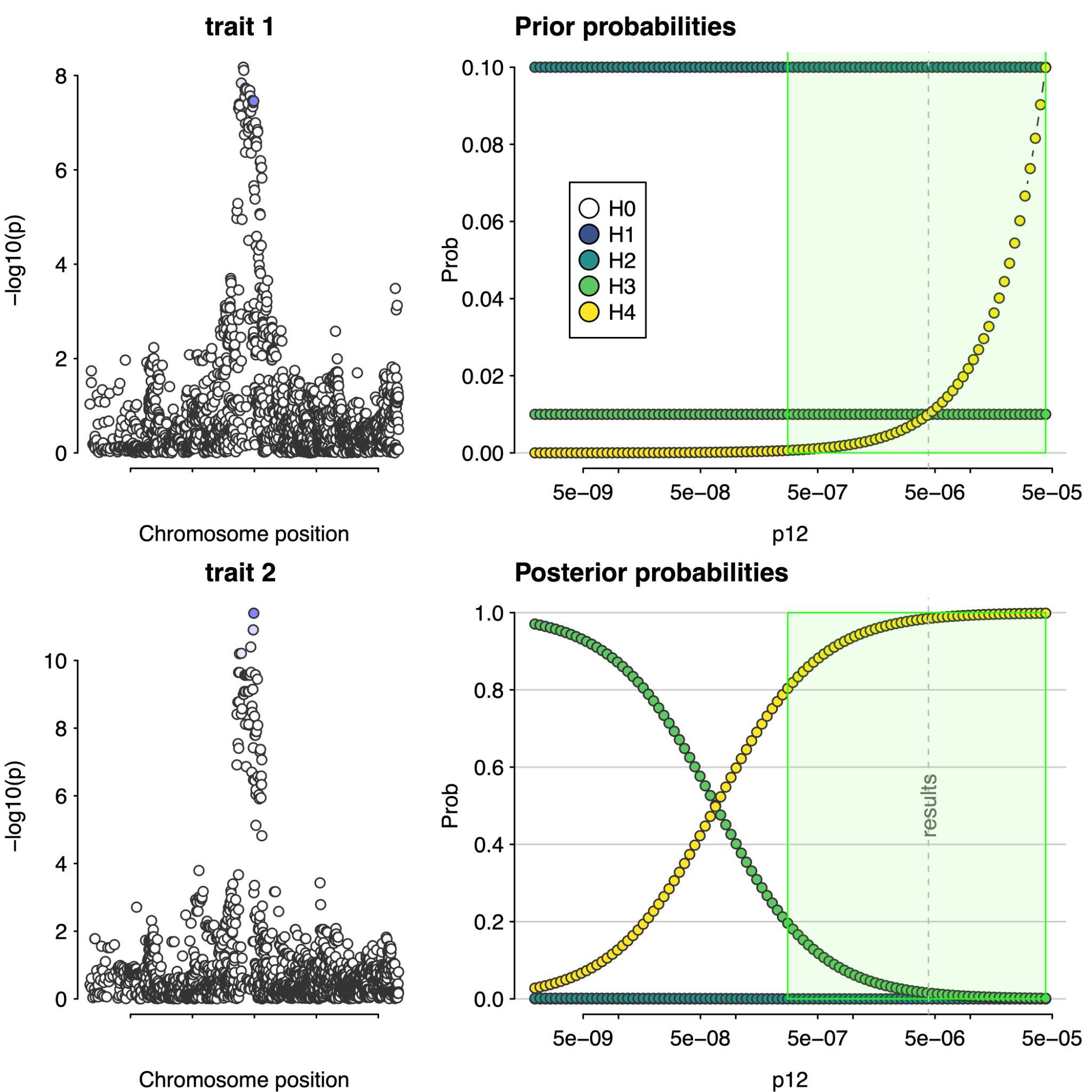


**Supplementary Figure 18: Colocalisation sensitivity analysis of rs174541 (2-linoleoyl-GPC) and *FADS1* expression.**

The left plots show the negative log-transformed *P*-values of every SNP within the 500 kb locus. The right plots show the prior and posterior probabilities of each of the five possible hypotheses for a given p12. The green box shows the range of p12 for which the colocalisation threshold (posterior probability of H4 > 0.8) is reached. The dashed line indicates the value of p12 chosen by the method described within the paper. Trait 1 is the metabolite exposure and trait 2 is the *FADS1* gene expression. H0-4 refer to the five possible hypotheses: H0, neither trait has a genetic association in the region; H1, only the exposure has a genetic association in the region; H2, only the outcome has a genetic association in the region; H3, both traits are associated, but with different causal variants; H4, the exposure and outcome are associated and share a single causal variant. Prob = probability. p12 is the prior probability that a given SNP within the locus is associated with both traits.


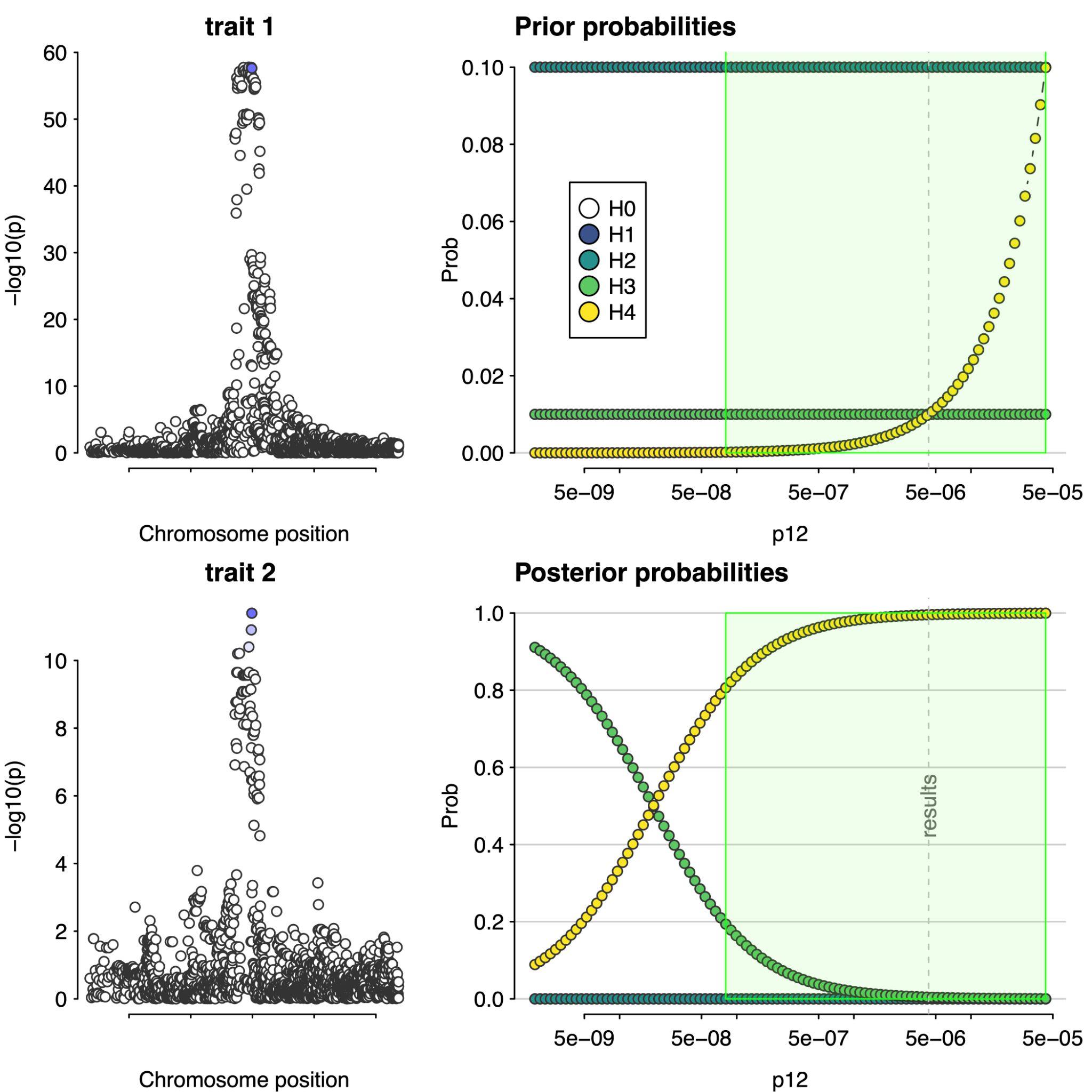


**Supplementary Figure 19: Colocalisation sensitivity analysis of rs174551 (1,2-dilinoleoyl-GPC) and *FADS1* expression.**

The left plots show the negative log-transformed *P*-values of every SNP within the 500 kb locus. The right plots show the prior and posterior probabilities of each of the five possible hypotheses for a given p12. The green box shows the range of p12 for which the colocalisation threshold (posterior probability of H4 > 0.8) is reached. The dashed line indicates the value of p12 chosen by the method described within the paper. Trait 1 is the metabolite exposure and trait 2 is *FADS1* gene expression. H0-4 refer to the five possible hypotheses: H0, neither trait has a genetic association in the region; H1, only the exposure has a genetic association in the region; H2, only the outcome has a genetic association in the region; H3, both traits are associated, but with different causal variants; H4, the exposure and outcome are associated and share a single causal variant. Prob = probability. p12 is the prior probability that a given SNP within the locus is associated with both traits.


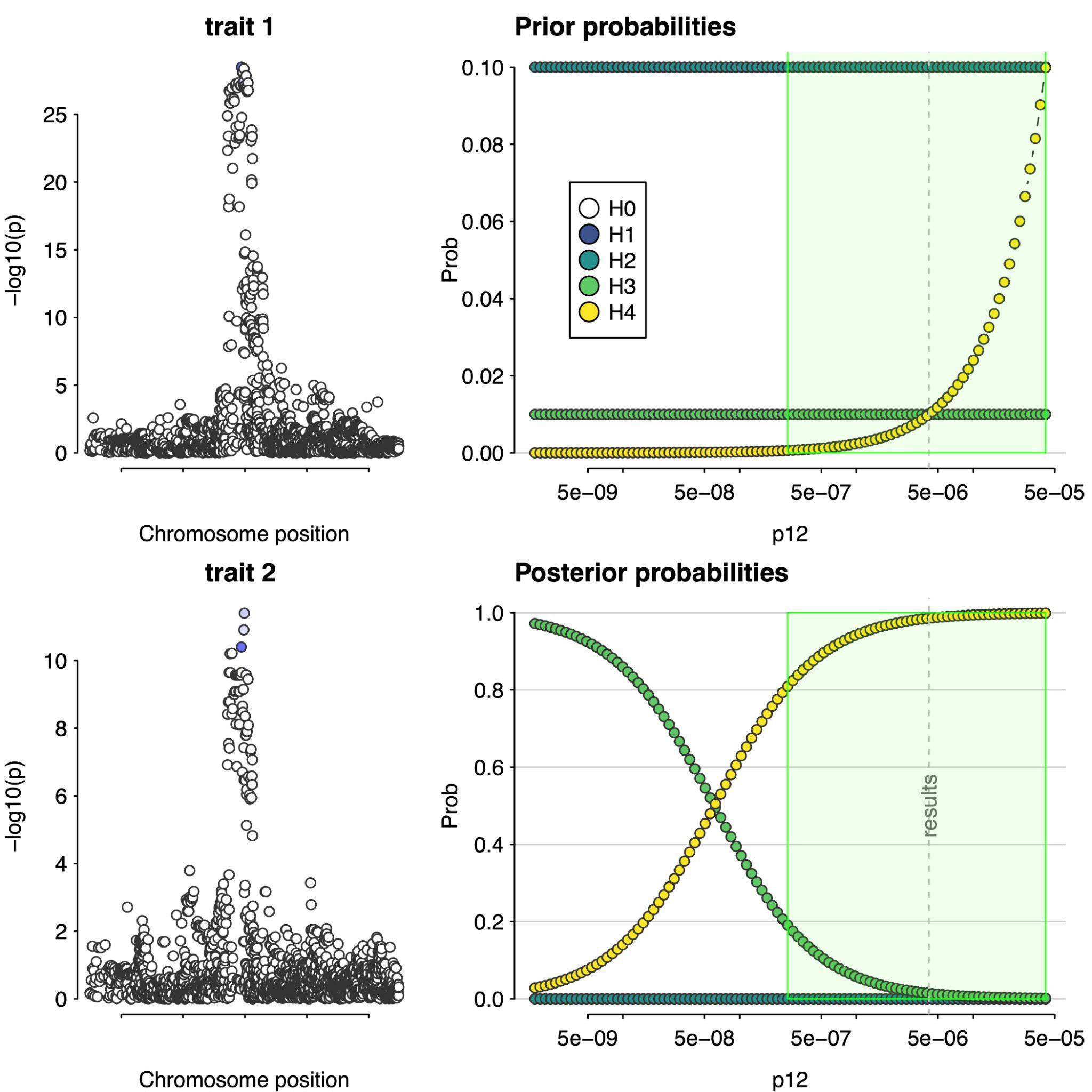


**Supplementary Figure 20: Colocalisation sensitivity analysis of rs99780 (1-pentadecanoyl-2-linoleoyl-GPC) and *FADS1* expression.**

The left plots show the negative log-transformed *P*-values of every SNP within the 500 kb locus. The right plots show the prior and posterior probabilities of each of the five possible hypotheses for a given p12. The green box shows the range of p12 for which the colocalisation threshold (posterior probability of H4 > 0.8) is reached. The dashed line indicates the value of p12 chosen by the method described within the paper. Trait 1 is the metabolite exposure and trait 2 is *FADS1* gene expression. H0-4 refer to the five possible hypotheses: H0, neither trait has a genetic association in the region; H1, only the exposure has a genetic association in the region; H2, only the outcome has a genetic association in the region; H3, both traits are associated, but with different causal variants; H4, the exposure and outcome are associated and share a single causal variant. Prob = probability. p12 is the prior probability that a given SNP within the locus is associated with both traits.
