# Supplementary material for: Mendelian randomisation analysis to discover plasma metabolites mediating the effect of obesity on cancer risk
Source: Br J Cancer. 2025 Sep 2;133(9):1344–53. doi: 10.1038/s41416-025-03170-7 (PMC12572173; doi:10.1038/s41416-025-03170-7)
Supplement: Supplementary file 3 — STROBE-MR Checklist [file 41416_2025_3170_MOESM3_ESM.pdf]

STROBE-MR checklist of recommended items to address in reports of Mendelian randomization studies[1 2]

Item No.	Section		Checklist item	Page No.	Relevant text from manuscript
1	**TITLE and ABSTRACT**		Indicate Mendelian randomization (MR) as the study's design in the title and/or the abstract if that is a main purpose of the study	1-2	"Mendelian randomisation analysis to discover plasma metabolites mediating the effect of obesity on cancer risk", "We screened 856 plasma metabolites to identify those associated with body mass index (BMI) and waist hip ratio (WHR), using Mendelian randomisation (MR)."
	**INTRODUCTION**				
2	**Background**		Explain the scientific background and rationale for the reported study. What is the exposure? Is a potential causal relationship between exposure and outcome plausible? Justify why MR is a helpful method to address the study question	3	"One approach to gain insight into the mechanistic basis of obesity-related cancer risk is to identify circulating metabolites mediating the effects of obesity.", "While studies have supported the influence of obesity on levels of several plasma metabolites, since levels reflect complex biological processes, observational studies can be biassed by confounding factors and reverse causation.", "Mendelian randomization (MR) is an analytical approach which seeks to address these biases."
3	**Objectives**		State specific objectives clearly, including pre-specified causal hypotheses (if any). State that MR is a method that, under specific assumptions, intends to estimate causal effects	3	"Here, we have used MR (adhering to STROBE-MR best practices8) in conjunction with mediation analysis to identify plasma metabolites mediating the effect of obesity on the risk of eight common cancers", "MR uses genetic variants as instrumental variables to evaluate the causal effects of exposures (risk factors) on outcomes."
	**METHODS**				
4	**Study design and data sources**		Present key elements of the study design early in the article. Consider including a table listing sources of data for all phases of the study. For each data source contributing to the analysis, describe the following:	4	"Figure 1 shows the overall study design", "Consortium details are provided in Supplementary Table 1"
		a)	Setting: Describe the study design and the underlying population, if possible. Describe the setting, locations, and relevant dates, including periods of recruitment, exposure, follow-up, and data collection, when available.	4	"GWAS meta-analysis data on individuals of European ancestry"
		b)	Participants: Give the eligibility criteria, and the sources and methods of selection of participants. Report the sample size, and whether any power or sample size calculations were carried out prior to the main analysis	4	"post-hoc power calculations provided in Supplementary Tables 2 and 3"

		c)	Describe measurement, quality control and selection of genetic variants	4	"The IVs for BMI/WHR were found using PLINK v.1.9 ($P < 5 \times 10{-}8$; $r2 < 0.01$, within a 0.5 Mb genomic region) to clump genome-wide significant single nucleotide polymorphisms (SNPs) with a minor allele frequency > 0.01 referenced to the 1000 Genomes Project European panel"
		d)	For each exposure, outcome, and other relevant variables, describe methods of assessment and diagnostic criteria for diseases	5	"For this analysis, we used summary cancer GWAS effect estimates from: (1) Online consortia resources, for breast and prostate cancer; (2) GWAS Catalog for ovarian, CRC, endometrial, and lung cancers; (3) Investigators of published work, for RCC and oesophageal cancer"
		e)	Provide details of ethics committee approval and participant informed consent, if relevant	4	"The study was undertaken using published GWAS data, hence ethical approval was not required."
5	**Assumptions**		Explicitly state the three core IV assumptions for the main analysis (relevance, independence and exclusion restriction) as well assumptions for any additional or sensitivity analysis	4	"Three core assumptions are made about the instrumental variables (IVs) – the IVs are associated with the exposure, there are no confounders of the IV-outcome associations, and the IVs only influence the outcome through the exposure."
6	**Statistical methods: main analysis**		Describe statistical methods and statistics used	4-6	"The effect of BMI and WHR on plasma metabolite levels was estimated using the inverse variance weighted random-effects (IVW-RE) model", "A Bonferroni-corrected P-value threshold was used to define statistical significance, adjusting for the number of harmonised metabolites.", "Using Cochran's Q statistic, we considered a P-value < 0.05 as being indicative of significant heterogeneity. To ensure robustness of any associations…", "We performed colocalisation analysis using the coloc R package v.5.2.329 to examine whether the genetically predicted metabolite level and cancer risk shared the same causal variant."
		a)	Describe how quantitative variables were handled in the analyses (i.e., scale, units, model)	4-6	"The effects were estimated as odds ratios (OR) per standard deviation (S.D.) increase in a metabolite level (ORSD), with a 95% confidence interval (CI)" and see above
		b)	Describe how genetic variants were handled in the analyses and, if applicable, how their weights were selected	4	See 4c

		c)	Describe the MR estimator (e.g. two-stage least squares, Wald ratio) and related statistics. Detail the included covariates and, in case of two-sample MR, whether the same covariate set was used for adjustment in the two samples	4	“The effect of BMI and WHR on plasma metabolite levels was estimated using the inverse variance weighted random-effects (IVW-RE) model”
		d)	Explain how missing data were addressed	5	“Data harmonisation and MR analyses were conducted using TwoSampleMR v.0.5.9, with SNPs not present in both the exposure and outcome GWAS removed.”
		e)	If applicable, indicate how multiple testing was addressed	5	“A Bonferroni-corrected P-value threshold was used to define statistical significance, adjusting for the number of harmonised metabolites.”
7	**Assessment of assumptions**		Describe any methods or prior knowledge used to assess the assumptions or justify their validity	8	“For all metabolites, the F-statistics, which is a measure of the strength of the association of IVs with an exposure, were greater than 59.8 for BMI and 45.8 for WHR; hence there was no evidence of weak instrument bias (defined by F-statistic < 10)”, “Conditional F-statistics and horizontal pleiotropy estimates were calculated using strength_mvmr and pleiotropy_mvmr, respectively, within MVMR v.0.4”
8	**Sensitivity analyses and additional analyses**		Describe any sensitivity analyses or additional analyses performed (e.g. comparison of effect estimates from different approaches, independent replication, bias analytic techniques, validation of instruments, simulations)	5-6	“Using Cochran’s Q statistic, we considered a P-value < 0.05 as being indicative of significant heterogeneity. To ensure robustness of any associations, MR analyses using the inverse variance weighted fixed-effects, maximum likelihood, simple median, weighted median, simple mode, and weighted mode were also performed. Leave-one-out analysis was also performed to detect outlying and pleiotropic SNPs”, “The MR-Egger intercept test was used to evaluate directional pleiotropy when three or more IVs were available”, “We also assessed reverse causation, wherein the effect of cancer may influence plasma metabolite levels, using bidirectional MR.”, “We performed colocalisation analysis using the coloc R package v.5.2.3”, “Metabolite GWAS data from the Canadian Longitudinal Study of Aging were used for replication of metabolite and cancer risk associations.”
9	**Software and pre-registration**				
		a)	Name statistical software and package(s), including version and settings used	4-7	“PLINK v.1.9 ($P < 5 \times 10^{-8}$; $r2 < 0.01$, within a 0.5 Mb genomic region)”, “TwoSampleMR v.0.5.9”,

			“using the coloc R package v.5.2.3 […] all SNPs within a +/-0.5 Mb region around the SNP acting as an IV for the metabolite were included. Based on advocated priors, the posterior probability of hypothesis 4 (H4) or PPshared (two traits sharing a single causal variant) > 0.8 was considered to provide evidence of colocalisation.”, “using the estimateSyy function in metaCCA v.1.13.2”
	b) State whether the study protocol and details were pre-registered (as well as when and where)	N/A	
	**RESULTS**		
10	**Descriptive data**		
	a) Report the numbers of individuals at each stage of included studies and reasons for exclusion. Consider use of a flow diagram	4	“the GIANT consortium and the UK Biobank (BMI, 681,275 samples; WHR, 697,734 samples)”, “the INTERVAL study, which quantified 867 plasma metabolites in individuals of European ancestry using Metabolon (726 metabolites) and Nightingale (141 metabolites) assays that measured plasma abundances in 8,153 and 37,359 individuals, respectively”
	b) Report summary statistics for phenotypic exposure(s), outcome(s), and other relevant variables (e.g. means, SDs, proportions)	4	“Consortium details are provided in Supplementary Table 1 and post-hoc power calculations provided in Supplementary Tables 2 and 3”
	c) If the data sources include meta-analyses of previous studies, provide the assessments of heterogeneity across these studies	N/A	
	d) For two-sample MR:<br>i. Provide justification of the similarity of the genetic variant-exposure associations between the exposure and outcome samples<br>ii. Provide information on the number of individuals who overlap between the exposure and outcome studies	5	“Since the UK Biobank was used to obtain genetic instruments for obesity traits, the CRC and oesophageal GWAS association statistics were recalculated from primary data excluding UK Biobank samples to avoid sample overlap bias”
11	**Main results**		
	a) Report the associations between genetic variant and exposure, and between genetic variant and outcome, preferably on an interpretable scale	4-5	“The IVs for BMI/WHR were found using PLINK v.1.9 ($P < 5 \times 10^{-8}$; $r^2 < 0.01$, within a 0.5 Mb genomic region) to clump genome-wide significant single nucleotide polymorphisms (SNPs) with a minor allele frequency > 0.01 referenced to the 1000 Genomes Project European panel (Supplementary Tables 4 and 5)”, “IVs of the

					metabolite levels were defined as in Stage 1 (Supplementary Table 7)”
		b)	Report MR estimates of the relationship between exposure and outcome, and the measures of uncertainty from the MR analysis, on an interpretable scale, such as odds ratio or relative risk per SD difference	8-9	e.g. “a per S.D. reduction in genetically predicted levels of three BMI-associated metabolites: 2-linoleoyl-GPC (ORSD = 0.58; 95% CI: 0.49-0.68), 1,2-dilinoleoyl-GPC (ORSD = 0.83; 95% CI: 0.76-0.91), 1-pentadecanoyl-2-linoleoyl-GPC (ORSD = 0.76; 95% CI: 0.70-0.82), and BMI- and WHR-associated 1-linoleoyl-GPC (ORSD = 0.60; 95% CI: 0.51-0.70) were associated with increased risk of CRC.”
		c)	If relevant, consider translating estimates of relative risk into absolute risk for a meaningful time period	N/A	
		d)	Consider plots to visualize results (e.g. forest plot, scatterplot of associations between genetic variants and outcome versus between genetic variants and exposure)	Figs. 2-4	
12	**Assessment of assumptions**				
		a)	Report the assessment of the validity of the assumptions	8-10	e.g. “We examined the robustness of these MR findings with a heterogeneity test, directional pleiotropy test, leave-one-out analysis, and reverse-causation test, retaining only those metabolites robustly influenced by BMI and WHR. We did not find significant heterogeneity for Bonferroni-significant metabolites (P < 0.05), apparent directional horizontal pleiotropy using the MR-Egger test (PEgger-intercept < 0.05), or a single SNP driving any of the associations from the leave-one-out analysis. To assess potential reverse causation, whereby the metabolites influenced BMI or WHR, we performed bidirectional MR using metabolite levels as the exposures and BMI or WHR as the outcome (Supplementary Tables 10 and 11)”
		b)	Report any additional statistics (e.g., assessments of heterogeneity across genetic variants, such as I^2, Q statistic or E-value)	8-10	e.g. “(Supplementary Table 19) […] Horizontal pleiotropy was also detected for all multivariable MR analyses.”
13	**Sensitivity analyses and additional analyses**				

		a)	Report any sensitivity analyses to assess the robustness of the main results to violations of the assumptions	8-10	See above
		b)	Report results from other sensitivity analyses or additional analyses	8-10	See above
		c)	Report any assessment of direction of causal relationship (e.g., bidirectional MR)	8-10	See above
		d)	When relevant, report and compare with estimates from non-MR analyses	N/A	
		e)	Consider additional plots to visualize results (e.g., leave-one-out analyses)	Fig. 4	
	**DISCUSSION**				
14	**Key results**		Summarize key results with reference to study objectives	12-13	“we identify potential mediators of obesity-driven cancer risk, with four plasma metabolites from univariable MR that were associated with both BMI/WHR and CRC risk and one metabolite associated with BMI and breast cancer”, “Herein we have been able to provide evidence for potential mediation of obesity-driven CRC and breast cancer risk by performing a metabolome-wide MR analysis”
15	**Limitations**		Discuss limitations of the study, taking into account the validity of the IV assumptions, other sources of potential bias, and imprecision. Discuss both direction and magnitude of any potential bias and any efforts to address them	12-13	“Although the F-statistics of all IVs used were above 10, indicating low risk of weak instrument bias, in seeking to minimise false positives by adopting a strict significance threshold using Bonferroni correction we may have filtered out some important metabolites. Our analyses were restricted to individuals of European ancestry to prevent confounding from population stratification. Hence, future studies are required to establish whether the same metabolites mediate the effect of obesity in populations of non-European ancestries. Finally, sex-specific effects could not be investigated due to the lack of sex-stratified data.”, “the wide confidence intervals reflect the limited predictive power of the metabolite IVs.”
16	**Interpretation**				
		a)	Meaning: Give a cautious overall interpretation of results in the context of their limitations and in comparison with other studies	13	“Herein we have been able to provide evidence for potential mediation of obesity-driven CRC and breast cancer risk by performing a metabolome-wide MR analysis.”, “Hence, our observations are in broad agreement with this study's findings, which

				reported a metabolite-mediating effect of 61% for BMI-driven CRC risk.”
		b) Mechanism: Discuss underlying biological mechanisms that could drive a potential causal relationship between the investigated exposure and the outcome, and whether the gene-environment equivalence assumption is reasonable. Use causal language carefully, clarifying that IV estimates may provide causal effects only under certain assumptions	11-12	“Accepting the limitations of the mediation analysis and although speculative, the omega-6 polyunsaturated fatty acid (PUFA) metabolic pathway may explain the CRC risk associations found by the MR analyses.”, “This makes it difficult to speculate on potential pathways by which obesity-driven breast cancer risk is mediated by the metabolome, due to the lack of menopausal-stratified breast cancer GWAS available for this study.”
		c) Clinical relevance: Discuss whether the results have clinical or public policy relevance, and to what extent they inform effect sizes of possible interventions	13	“the ability of aspirin to irreversibly inhibit COX-1 and COX-2 and lower proinflammatory signals for CRC chemoprevention”
17	**Generalizability**	Discuss the generalizability of the study results (a) to other populations, (b) across other exposure periods/timings, and (c) across other levels of exposure	12	“A study using a menopausal-stratified breast cancer GWAS may therefore detect mediators, which we unfortunately were not empowered to identify.”, “Our analyses were restricted to individuals of European ancestry to prevent confounding from population stratification. Hence, future studies are required to establish whether the same metabolites mediate the effect of obesity in populations of non-European ancestries. Finally, sex-specific effects could not be investigated due to the lack of sex-stratified data.”
	**OTHER INFORMATION**			
18	**Funding**	Describe sources of funding and the role of funders in the present study and, if applicable, sources of funding for the databases and original study or studies on which the present study is based	14-15	See “Funding” heading
19	**Data and data sharing**	Provide the data used to perform all analyses or report where and how the data can be accessed, and reference these sources in the article. Provide the statistical code needed to reproduce the results in the article, or report whether the code is publicly accessible and if so, where	14	See “Data availability” heading
20	**Conflicts of Interest**	All authors should declare all potential conflicts of interest	14	See “Conflict of interest” heading

This checklist is copyrighted by the Equator Network under the Creative Commons Attribution 3.0 Unported (CC BY 3.0) license.

1. Skrivankova VW, Richmond RC, Woolf BAR, Yarmolinsky J, Davies NM, Swanson SA, et al. Strengthening the Reporting of Observational Studies in Epidemiology using Mendelian Randomization (STROBE-MR) Statement. JAMA. 2021;under review.

2. Skrivankova VW, Richmond RC, Woolf BAR, Davies NM, Swanson SA, VanderWeele TJ, et al. Strengthening the Reporting of Observational Studies in Epidemiology using Mendelian Randomisation (STROBE-MR): Explanation and Elaboration. BMJ. 2021;375:n2233.
